# Targeting p75NTR activity alleviates the neurotoxic effect of high glucose on iPSC-derived dopaminergic neurons

**DOI:** 10.1186/s13287-026-04965-y

**Published:** 2026-03-21

**Authors:** Konstantina Chanoumidou, Ioanna Zota, Maria Anna Papadopoulou, Chrystalla Konstantinou, Alexandros Tsimpolis, Electra Tsagliotis, Maria Tziortziou, Katerina Ntarntani, Anne Grünewald, Matthieu David Lavigne, Achille Gravanis, Ioannis Charalampopoulos

**Affiliations:** 1 Department of Pharmacology, Medical School, University of Crete, 71003 Heraklion, Greece; 2 Institute of Molecular Biology and Biotechnology, Foundation for Research and Technology-Hellas, 71003 Heraklion, Greece; 3 Luxembourg Centre for Systems Biomedicine, University of Luxembourg, 4362 Esch-sur-Alzette, Luxembourg

**Keywords:** iPSC, p75NTR, Neurotrophins, Dopaminergic neurons, Glucotoxicity, Neurodegeneration, Neuroinflammation

## Abstract

**Background:**

Hyperglycemia, a hallmark of diabetes mellitus, is a metabolic condition that highly affects the nervous system. While evidence from epidemiological and animal studies links diabetes to dopaminergic dysfunction and an increased risk of Parkinson’s disease, the underlying mechanisms remain unclear. Here, we examined the effects of high glucose on human iPSC-derived dopaminergic neurons and glial cells to better understand the pathogenic alterations that lead to neurotoxicity. Previous implication of neurotrophins in the neurological manifestations of diabetes prompted us to focus on the role of p75NTR neurotrophin receptor (p75NTR) in dopaminergic neurodegeneration under hyperglycemic conditions.

**Methods:**

iPSC-derived dopaminergic neurons, astrocytes and microglia were treated with high glucose (50mM, 100mM) for 48 h to simulate hyperglycemia. Cytotoxicity assays, RNA sequencing and DNA damage assessments were employed to investigate the pathological alterations induced by high glucose exposure in neurons. Pharmacological targeting of p75NTR activity allowed investigation of its involvement in glucose neurotoxicity. Glial-mediated neurotoxicity was evaluated using conditioned media and inflammatory marker analysis.

**Results:**

High glucose treatment led to DNA damage, activation of JNK signaling and cell death in neurons. Importantly, we observed upregulation of p75NTR and its pro-apoptotic ligand pro-NGF, suggesting activation of the pro-NGF/p75NTR axis in high glucose-treated neurons. Inhibition of p75NTR activity rescued neuronal cell death, identifying p75NTR as a central mediator of glucose neurotoxicity. Furthermore, glucose overload sensitized neurons to 6-hydroxydopamine (6-OHDA), increasing their vulnerability to neurotoxic insults—an effect reversed by p75NTR blockade. Treatment with BNN27, a synthetic NGF mimetic, prevented neuronal loss through p75NTR and TrkA receptors, suggesting neurotrophin signaling as a potential therapeutic target for combating high glucose-induced neuronal damage. Finally, we demonstrated the contribution of glial cells to neurodegeneration since high glucose treatment of iPSC-derived astrocytes and microglia enhanced their inflammatory potential and triggered the release of neurotoxic factors, causing pro-apoptotic effects on neurons.

**Conclusions:**

Our findings show that high glucose impairs human dopaminergic neuron survival through activation of the pro-NGF/p75NTR axis and indirect glia-mediated mechanisms. Targeting p75NTR signaling may offer neuroprotective benefits in diabetes-related neurodegeneration, particularly for patients at risk of Parkinson’s disease.

**Graphical Abstract:**

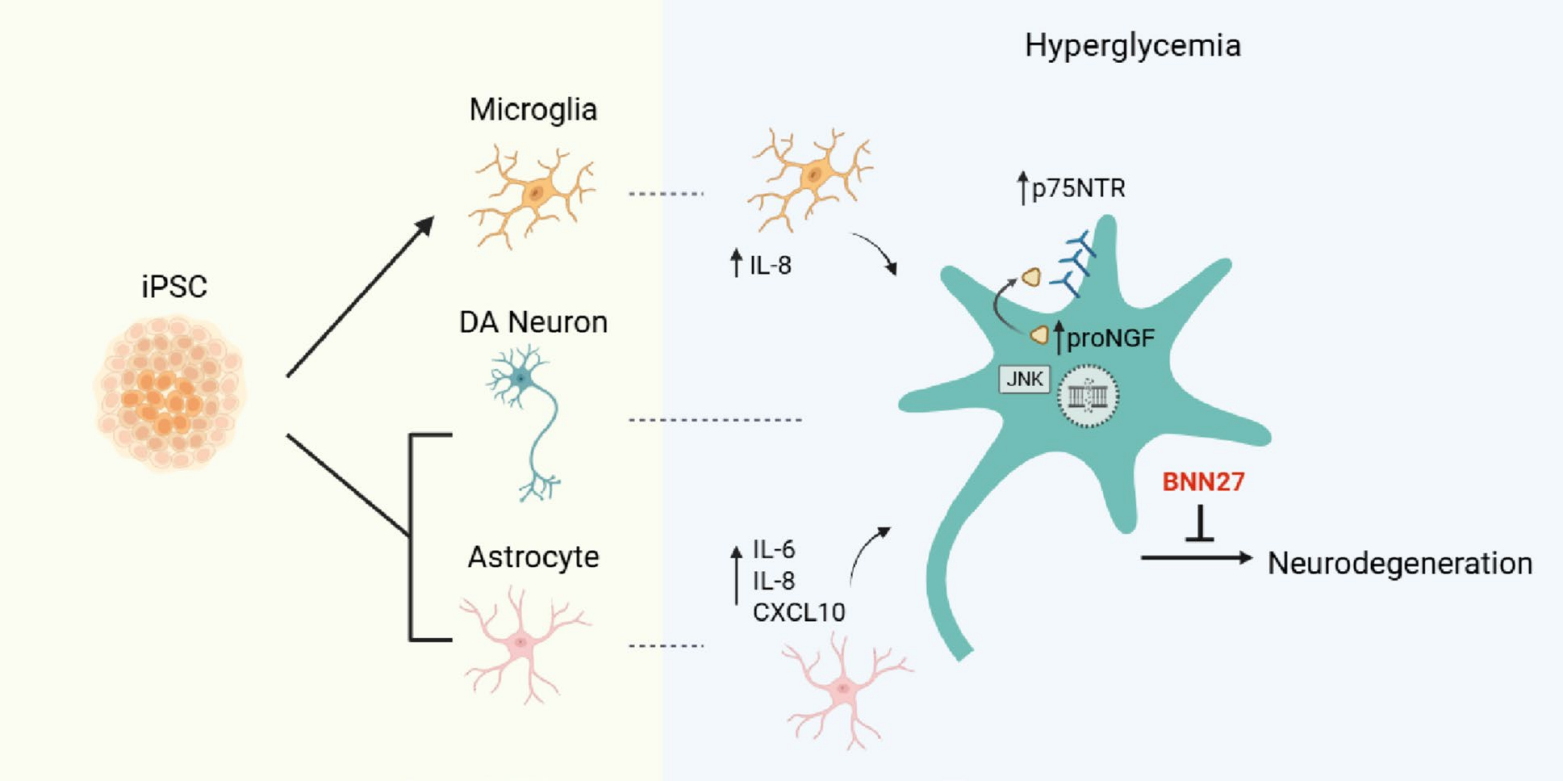

**Supplementary Information:**

The online version contains supplementary material available at 10.1186/s13287-026-04965-y.

## Introduction

Hyperglycemia, a defining feature of diabetes mellitus (DM), is a metabolic condition that highly affects the nervous system accelerating neurodegeneration. Although the connection between DM and Alzheimer’s disease is well documented [9], its association with Parkinson’s Disease (PD) is still largely unclear. Growing evidence links DM to dopaminergic neurodegeneration and increased risk of PD [10, 21, 46, 89]. Animal models and patients with DM show striatal dopaminergic dysfunction, altered dopamine neurotransmission and have increased risk for parkinsonian symptoms [23, 47, 68]. However, the mechanistic interlink between the two disorders remains unclear impairing the development of neuroprotective therapies.

Glucose is the main source of energy for the brain. Both preclinical and clinical evidence suggest that elevated glucose levels can have detrimental effects on neurons. Preclinical studies have demonstrated that high glucose leads to neuronal injury, synaptic dysfunction and changes in the brain [58, 77, 92]. These findings are supported by clinical studies linking hyperglycemia and DM with brain atrophy, reduced cortical thickness and increased risk of dementia [22, 59, 63]. Glucose neurotoxicity has been linked to mitochondrial dysfunction, oxidative stress and accumulation of AGEs [84], mechanisms that are also common with many neurodegenerative disorders. Additionally, diabetes leads to systemic inflammation, changes in blood brain barrier (BBB) integrity and gliosis in the brain [50]. Streptozotocin (STZ)-treated mice display increased brain sensitivity to peripheral LPS-induced inflammation [50] while STZ treatment in the rotenone model of PD activates microglia and eventually worsens neurodegeneration and motor symptoms [90].

Neurotrophins, a major class of endogenous neuroprotective molecules, have been associated with the neurological manifestations of diabetes [36, 37]. The pro-apoptotic NGF isoform, pro-NGF, is up-regulated in diabetic retinopathy [25, 61], while BDNF protects hippocampal neurons from hyperglycemia-driven apoptosis [91]. The neuroregenerative properties of neurotrophin signaling indicate neurotrophin receptors as promising therapeutic targets for neuroprotection. The p75 neurotrophin receptor (p75NTR), a member of the TNF receptor superfamily, binds all mature and pro-neurotrophins. While best known for mediating pro-apoptotic signaling upon binding to pro-neurotrophins under pathological conditions [44, 48], p75NTR can also promote cell survival in a ligand- and context-dependent manner [14, 56]. Notably, p75NTR is up-regulated in peripheral diabetic neuropathy [20, 38, 78] while in STZ-treated rodents the pro-NGF/p75NTR signaling promotes Blood-Retina-Barrier disruption and neuroinflammation in retina [26, 61]. However, its role in brain pathology under diabetic conditions is yet unexplored.

To date, all studies investigating the impact of hyperglycemia on the dopaminergic system have been conducted in animal models. Leveraging the technology of human induced Pluripotent Stem Cells (hiPSCs), we examined the effects of high glucose (HG) on hiPSC-derived dopaminergic (DA) neurons, astrocytes and microglia to gain deeper insights into the pathogenic mechanisms of hyperglycemia—induced neurodegeneration and the contribution of glial cells in this pathology in a human relevant model. hiPSC-derived cells enable recapitulation of disease relevant mechanism for molecular interrogation [87]. We focused on the role of p75NTR in glucose neurotoxicity and evaluated p75NTR as a potential target for intervention. Our findings show that HG primarily induces DNA damage and neuronal loss. RNA sequencing analysis confirmed the induction of numerous stress-related processes including p53 signaling, response to TNF and DNA damage repair. The accumulation of pro-NGF and p75NTR in HG-treated neurons suggests induction of the pro-apoptotic pro-NGF/p75NTR axis in hyperglycemic conditions. Notably, inhibition of p75NTR prevented neuronal cell death, highlighting p75NTR as a potential therapeutic target for mitigating glucotoxicity in dopaminergic neurons. Glucose overload increased the vulnerability of DA neurons to 6-OHDA - induced cytotoxicity while inhibition of p75NTR activity significantly reduced this effect. We tested BNN27, a synthetic NGF mimetic which targets p75NTR and TrkA receptors, against HG-induced toxicity and we highlight its neuroprotective action in hyperglycemic condition. Finally, we showed that HG increases astrocyte and microglia responsiveness to pro-inflammatory stimuli and promotes the release of neurotoxic factors showcasing the critical involvement of glial cells in hyperglycemia-induced neurodegeneration.

## Materials and methods

### Cell lines

Three healthy human iPSC lines (SFC856-03-04, SFC841-03-01, SBAD-03-01) were kindly provided by Dr Μ. Z. Cader which were reprogrammed as part of IMI StemBANCC [60]. iPSCs were reprogrammed from skin fibroblasts of three healthy control individuals. These cell lines were used for the generation of neural progenitor cells, dopaminergic neurons and astrocytes. Microglia were differentiated from a healthy iPSC line derived from a female donor provided by Anne Grünewald. All cell lines have been tested and were negative for mycoplasma contamination.

### Generation of neural progenitor cells (NPCs)

Neural progenitor cells were generated as previously described [73]. Briefly, iPSCs were cultured on mouse embryonic fibroblasts (MEFs) until they form dense colonies in hESC medium (DMEM-F12 medium (Gibco, 21331-020), 20% KO serum replacement (Gibco, 10828028), Non-essential amino acids (Gibco, 11-140-050), Pen/Strep (Gibco, 15140122), l-Glutamine (Gibco, A2916801), 2-Mercaptoethanol (Gibco, 31350010) supplemented with 5 ng/ml FGF2 (Peprotech, 100-18C). Next, iPSC colonies were cut into small pieces and detached from MEFs via treatment with 2 mg/mL collagenase IV (Sigma, C1764) for 15–30 min at 37 °C. Cells were collected and resuspended in human embryonic stem cell (hESC) medium without FGF2 supplemented with 1 µM Dorsomorphin (Abcam, ab120843), 3 µM CHIR99021 (Sigma, SML1046), 10 µM SB-431542 (Stem Cell Technologies, 72232) and 0.5 µM Purmorphamine (Stem Cell Technologies, 72202). Embryoid bodies (EBs) were formed by culturing cells in non-adherent petri dishes (Corning) for six days. On the second day, medium was changed to N2B27 medium (1:1 Neurobasal (Gibco, 21103-049) and DMEM-F12 medium (Gibco, 21331-020), 1:100 B27 supplement lacking vitamin A (Gibco, 12587010), 1:200 N2 supplement (Gibco, 17502048), 1% penicillin/streptomycin (Gibco, 15140122) supplemented with the same factors as on Day 0. On day 4, dorsomorphin and SB-431542 were removed, whereas 150 µM l-Ascorbic acid (Sigma, A4544) was added to the medium. On day 6, EBs were partially broken into smaller pieces via pipetting and plated on Matrigel- (Matrigel Growth-factor-reduced, Corning, 354263) coated 12-well plates. When confluent, NPCs were passaged with Accutase (Sigma, A6964). After three passages, purmorphamine was replaced by 0.5 µM SAG (Abcam, ab142160). NPCs were expanded until passage 6 and then could be frozen or subcultured in NPC medium (N2B27 medium supplemented with 0.5 µM SAG, 3 µM CHIR, 150 µM Ascorbic acid). NPC identity was evaluated with immunostaining for Nestin. NPCs were used for neuronal differentiation after passage 8.

### Differentiation of NPCs towards dopaminergic neurons and glucose treatment

NPCs were seeded at density of 36,000 cells/cm^2^ on Matrigel-coated 12-well plate format in NPC medium (Day -1). On Day 0, the medium was replaced by N2B27 medium containing 1 μm SAG, 75 µM AA, 2 ng/mL BDNF (Peprotech, 450-02) and 2 ng/mL GDNF (Peprotech, 450-10). On day 6, the medium was replaced by N2B27 medium containing 75 µM AA, 2 ng/mL BDNF, 2 ng/mL GDNF, 1 ng/mL TGF-β3 (Peprotech, 100-36E) and 100 µM dbcAMP (Sigma, D0627). 5 ng/mL Activin A (Stem Cell Technologies, 78001) was added to the medium from day 6 to day 10. Cells were split in ratio 1:3 on day 8 of differentiation. Neuronal identity was confirmed with immunostaining for the neuronal markers TUJ1, MAP2 and the dopaminergic marker TH on day 21. The percentage of TUJ1 + cells was > 90% at day 21. Neurons were cultured in N2B27 medium containing 20mM d-glucose. This glucose concentration was considered as the control condition. Neurons were exposed to 50mM or 100mM d-glucose (Sigma, G8769) for 48 h (from day19 to day 21 of differentiation) to mimic hyperglycemia.

### Generation of astrocytes, stimulation and glucose treatment

For the generation of human iPSC-derived astrocytes, we followed the protocol originally described by Perriot et al. [69]. On Day 0, iPSC colonies were cut and transferred to low-binding 6-well plates in NPC induction medium DMEM/F-12 (Gibco, 21331-020), Glutamax (Gibco, 3505006), Pen/Strep (Gibco, 15140122), 1x N2 (Gibco, 17502048), 1x B27 w/o vitamin A (Gibco, 12587010), 500 ng/ml Noggin (Peprotech, 120-10c), 20 µM SB-431,542 (Stem Cell Technologies, 72232), 4 ng/ml FGF-2 (Peprotech, 100-18C), 2µg/ml Laminin (Sigma-Aldrich, L2020) in order to form neural spheres. After 6–8 h, the generated spheres were transferred to poly-l-ornithine (Sigma, P4957)/Laminin-(Sigma-Aldrich, L2020)-coated 6-well plates in order to attach and form neural rosettes. The medium was changed every other day. On day 10, the medium was switched to NPC expansion medium consisting of DMEM/F-12 (Gibco, 21331-020), Glutamax (Gibco, 3505006), N2 (Gibco, 17502048), B27 w/o vitamin A (Gibco, 12587010), 10 ng/ml FGF-2 (Peprotech, 100-18C) and 10 ng/ml EGF (R&D, 236-EG). Once neural rosettes were formed (around day13), the STEMdiff™ Neural Rosette Selection Reagent (Stem Cell Technologies, 05832) was used to isolate them. The rosettes were then transferred to poly-l-ornithine/Laminin-coated plates and were further cultured in NPC expansion medium. The cells were split using TrypLE (Gibco, 12605010). After 6–8 passages, homogenous SOX2+ and PAX6+ cell populations of NPCs were generated. For the astrocyte differentiation (50.000 cells/cm^2^), NPCs were plated on Matrigel in Astrocyte induction medium containing DMEM/F-12 (Gibco, 21331-020), Glutamax (Gibco, 3505006), N2 (Gibco, 17502048), B27 w/o vitamin A (Gibco, 12587010), 10 ng/ml EGF (R&D, 236-EG) and 10 ng/ml LIF (Peprotech, 300-05). Medium changes were performed every other day and the cells were passaged with TrypLE (Gibco, 12605010) when confluent. On Day 14, the medium was changed to Astrocyte medium containing DMEM/F-12 (Gibco, 21331-020), Glutamax (Gibco, 3505006), B27 w/o vitamin A (Gibco, 12587010) and 20 ng/ml CNTF (Peprotech, 450-13). From Day 14 to Day 28, the cells were passaged when confluent in seeding density 40,000 cells/cm^2^ onto Matrigel-coated flasks. From Day 29 to Day 42, the cells were passaged when confluent in seeding density 25,000 cells/cm^2^. At Day 44, we obtained > 90% S100β + astrocytes. In this study, astrocytes of differentiation day 50 were used for experiments. Astrocyte medium contains 17.5mM d-glucose. This glucose concentration was considered as the control level. Astrocytes were exposed to high glucose 100mM d-glucose (Sigma, G8769) for 48 h to generate hyperglycemic conditions. Cell exposure to 10 ng/ml IL-1β beta (Peprotech, 200-01B) and 30 ng/ml TNFa (Peprotech 300-01A) was used to activate astrocytes.

### Generation, stimulation of microglia and glucose treatment

Embryonic like macrophage precursors were generated from human iPSCs as previously described by [33, 86]. Briefly, 4 × 10^6^ iPSCs were seeded into an Aggrewell 800 well (STEMCELL Technologies, 34850) to form EBs, in EB medium containing mTESR (Stem Cell Technologies, 100-0276), 50 ng/mL BMP4 (Invitrogen, PHC9534), 50 ng/mL VEGF (Invitrogen, PHC9394), and 20 ng/mL SCF (Miltenyi, 130-096-695) supplemented with 10 µM Rock inhibitor (Abcam Biochemicals, Ab120129) on the day of plating. Medium was changed daily. After four days, EBs were harvested and transferred to a 6 well low attachment plate, where they were further cultured for 3 days in EB medium. Then, EBs were collected and transferred to a T175 flask in X-VIVO15 (Lonza, LZBE04-4418 F) medium supplemented with 100 ng/mL M-CSF (Invitrogen, PHC9501), 25 ng/mL IL-3 (Invitrogen, PHC0033), 2 mM Glutamax (Gibco, 35050), 100 U/mL penicillin and 100 µg/mL streptomycin (Gibco, 15140122), and 0.055 mM β-mercaptoethanol (Gibco, 31350010). 10 ml fresh medium was added weekly. After four weeks in culture, macrophage precursors were collected from the supernatant, were strained (40 μm, Corning) and plated (0.8 × 10^6^ cells per well of a tissue culture 6-well plate) for terminal differentiation towards microglia. Cells were cultured in Microglia medium containing Advanced DMEM/F12 (Invitrogen), 0.055 mM β-mercaptoethanol (Gibco, 31350010), 10 ng/ml GM-CSF (Peprotech, 300-03), 100 ng/ml IL-34 (Peprotech, 200-34), 25 ng/ml M-CSF (Invitrogen) and 50 ng/ml TGFβ1 (Peprotech) for 2 weeks and medium was changed every other day. Microglia medium contains 17.5mM d-glucose. Microglia were treated with 100mM d-glucose (Sigma, G8769) for 48 h to generate the hyperglycemic condition and 100 ng/ml LPS (Invitrogen, 00497693) during the last 6 h to activate cells.

### Immunocytochemistry

Cells were fixed with 4% paraformaldehyde for 15 min. Following three washes with 1xPBS (Gibco, 14190094), cells were treated with 0.1% Triton X-100 for 5 min. Next, the cells were incubated with blocking solution containing 10% serum, 0.3% Triton X-100 and 0.1% BSA (Gibco, 15260037) in 1× PBS for 1 h at RT. Primary antibodies were added in a buffer containing 1% serum and 0.1% Triton-X in 1xPBS overnight at 4 °C. Upon three washes with 1xPBS, the secondary antibodies were added in 1xPBS at RT for 1 h. Cell nuclei were visualized with Hoechst (H3570, Invitrogen). Images were obtained using a Confocal Laser Scanning Microscope Leica TCS SP8. Representative images are presented in the Figures. Primary antibodies are shown in Table 1.

**Table 1:** Primary antibodies for immunocytochemistry and western blot

Antibody	Company	Cat.
TUJ1	Biolegend	801,201
MAP2	Merck	MAB3418
TH	Abcam	Ab137721
S100β (E7C3A)	Cell Signaling	#90393
GFAP	Merck	ab5541
ΕΑΑΤ1	Abcam	Ab416
IBA1	Wako	019-19741
Synapsin I	Abcam	Ab254349
Synaptophysin	Merck	MAB5258
Phospho-Histone H2A.X (Ser139)	Merck	05-636
TrkA	Merck	06-574
TrkB	Merck	07-225-I
p75NTR	Biolegend	83,701
pJNK	Cell Signaling	4668
tJNK	Cell Signaling	9252
Beta Actin	Santa Cruz Biotechnology	sc-47778

### l-Glucose and mannitol treatments

To investigate the induction of osmotic stress, neurons were treated with 100 mM l-glucose (Sigma, G5500), 100 mM mannitol (Sigma, M4125), or 100 mM d-glucose (Sigma, G8769) for 48 h.

### Inhibition of p75NTR and sortilin activity

To inhibit p75NTR activity, neurons were treated with 2.5 ng/ml p75NTR MC-192 (Abcam, ab6172) or 400nM LM11A-31 (MedChemExpress, HY-117088) for 48 h. For sortilin inhibition, neurons were treated with 1 µM AF38469 (MedChemExpress, HY-12802) for 48 h.

### Amyloid-β treatment

The Amyloid-β (1–42) peptide was purchased from AnaSpec (AS-20276, AnaSpec, Fremont, CA, USA) and oligomers were prepared according to the manufacturer’s instructions and previously described protocols [45, 51]. Peptides were diluted in Neurobasal Medium (Gibco, 21103-049) at the indicated concentrations. Neurons were treated with 10µΜ Amyloid-β (1–42) for 48 h.

### 6-OHDA treatment

6-Hydroxydopamine hydrochloride (6-OHDA) was dissolved according to manufacturer’s instructions (Sigma, H4381). Neurons were treated with 50 µM 6-OHDA for 16 h to induce oxidative stress.

### BNN27 treatment

BNN27 was initially dissolved in DMSO to prepare a stock solution of 100mM. Next, cell culture medium was used to generate an intermediate dilution of 1mM. The solution was incubated for 10 min at 37 °C. Finally, neurons were treated with 1 µM BNN27 for 48 h and the effect on cell viability was evaluated with the Celltox assay (Promega, G8742).

### Celltox cytotoxicity assay

Celltox assay (G8742, Promega Corporation, Maddison, WI, USA) was used according to the manufacturer’s instruction. Celltox reagent was added to the cells 24 h before imaging. Hoechst (H3570, Invitrogen) was used for nuclei labeling. Cells were imaged with a Leica DM IL LED microscope. The percentage of dead cells was determined by normalizing the count of TUNEL^+^ cells to the total number of Hoechst^+^ cells. Analysis of TUNEL^+^ and Hoechst^+^ cell numbers was performed using the FIJI software. Representative images are presented in the Figures.

### TUNEL assay

Cells were fixed with 4% paraformaldehyde and labeled with terminal deoxynucleotidyl transferase dUTP nick-end labeling (TUNEL, Roche, Hertfordshire, UK) following the manufacturer’s protocol. Cell nuclei were visualized by Hoechst (H3570, Invitrogen). Cells were imaged with a Leica DM IL LED microscope. The percentage of apoptotic neurons was determined by normalizing the count of TUNEL^+^ cells to the total number of Hoechst^+^ cells. Analysis of TUNEL^+^ and Hoechst^+^ cell numbers was performed using the FIJI software. Representative images are presented in the Figure S2.

### Cell lysis and western blotting

Cells were solubilized with Pierce™ IP Lysis Buffer (87788, Thermo Fischer Scientific, Waltham, MA, USA) supplemented with protease inhibitors (539138, Calbiochem, Darmstadt, Germany) and phosphatase inhibitors (524629, Calbiochem, Darmstadt, Germany). Total proteins were separated by electrophoresis on SDS-PAGE gels and were transferred to nitrocellulose membranes (Cytiva, GE10600002). After blocking with 5% w/v bovine serum albumin (BSA) (Applichem, A1391) in 1xTBST, membranes were incubated overnight at 4^ο^C with gentle shaking with the primary antibodies in 5% w/v BSA in 1xTBST. The membranes were incubated for 1 h with HRP-conjugated secondary antibodies in 5% w/v BSA in 1xTBST at RT and immunoblots were developed using the ECL Western Blotting Kit (Thermo Fisher Scientific). Image analysis and quantification of band intensities were performed with ImageJ Software. Primary antibodies are shown in Table 1. Full Western Blot Images are provided in Figures S4, S5.

### RNA-seq library preparation and differential expression analysis

#### Isolation of RNA and 3′ RNA sequencing

Dopaminergic neurons were treated with 100mM d-glucose for 48 h at day 19 of cell differentiation. We collected RNA from three independent cell differentiation experiments of one iPSC line (SFC856-03-04), considering them as three biological replicates. Total RNA was extracted using Trizol reagent (Thermo Scientific) as per the manufacturer’s protocol. The quantity and quality of extracted RNA samples were analyzed using RNA 6000 Nano kit on a bioanalyzer (Agilent). 500 ng of total RNA samples with RNA integrity number (RIN) > 7 were used for library construction using the 3′ mRNA-Seq Library Prep Kit FWD for Illumina (QuantSeq-LEXOGEN) as per the manufacturer’s instructions. Amplification was controlled by qPCR for obtaining optimal unbiased libraries across samples by assessing the number of cycles (15) required for amplification of the library. DNA High Sensitivity Kit for bioanalyzer was used to assess the quantity and quality of libraries, according to the manufacturer’s instructions (Agilent). Libraries were multiplexed and sequenced on an Illumina Nextseq 500 at the genomics facility of IMBB FORTH according to the manufacturer’s instructions.

### Differential expression analysis (DEA) and gene ontology (GO) enrichment analysis

Essentially as detailed previously [15], the quality of the FASTQ files was assessed with the FastQC software.^,^ Reads were aligned to the human (hg38) genome with Hisat2 (hisat2 -p32 -x $REFERENCE_GENOME -q fastq/$FILE_ID.fastq -S $FILE_ID.sam --score-min L,0,-0.5 -k 2). Due to low quality of RNA and sequencing data generating poor alignment efficiency for one of the biological replicates of the High Glucose (100 mM d-glucose) condition, we kept only two replicates for this condition in the subsequent steps of the analysis. Htseq-counts was utilized to summarize reads at the gene level (htseq-count -f bam syesigene_idbam/$FILE_ID.bamdata/refs/Homo_sapiens/UCSC/hg38/Annotation/Genes/genes.gtf>$COUNTS_DIR/NGS$FILE_ID). Differentially expressed genes (DEGs) between the control (20mM) and High Glucose (100 mM) condition were identified by running EdgeR through SARTools R wrapper with batch (experiment date) effect correction. A significance threshold of an adjusted p-value < 0.05 was applied. Functional enrichment analysis was performed using Metascape. Volcano and bidirectional bar plots were created in R with custom in-house scripts (available upon request).

### Quantitative RT-PCR

Total RNA was extracted from cells using Nucleozol (Macherey Nagel, 740404200). 1500ng RNA was reversely transcribed to cDNA using the High-Capacity cDNA Reverse Transcription Kit (ThermoFisher Scientific 4368814). qRT-PCR was carried out using the ΚΑPA SYBR FAST qPCR Master Mix (KAPA, KK4602) and gene expression was normalized to *Beta-Actin*. The primers are presented in Table 2.

**Table 2:** List of primers for qPCR

Gene	Forward primer	Reverse primer
*IL-6*	5ʹ-ACTCACCTCTTCAGAACGAATTG-3ʹ	5ʹ-CCATCTTTGGAAGGTTCAGGTTG-3ʹ
*Cxcl10*	5ʹ-GTGGCATTCAAGGAGTACCTC-3ʹ	5ʹ-TGATGGCCTTCGATTCTGGATT-3ʹ
*IL-8*	5ʹ-GGTGCAGTTTTGCCAAGGAG-3ʹ	5ʹ-TTCCTTGGGGTCCAGACAGA-3ʹ
*Ccl5*	5ʹ-TCATTGCTACTGCCCTCTGC-3ʹ	5ʹ-TACTCCTTGATGTGGGCACG-3ʹ
*Beta-Actin*	5ʹ-CCAACCGCGAGAAGATGAC-3ʹ	5ʹ-TAGCACAGCCTGGATAGCAA-3ʹ

### ELISA

Pro-NGF and pro-BDNF protein levels were quantified in the cell lysis and the supernatant of neurons cultured in 12 well plate format (one well per condition) by using the human pro-NGF and the human pro-BDNF Rapid ELISA kits (Biosensis, BEK-2226-1P/2P for pro-NGF and BEK-2237-2P for pro-BDNF), respectively. Briefly, the cell supernatant was collected, supplemented with protease inhibitors (539138, Calbiochem, Darmstadt, Germany) and centrifuged for 5 min at 10,000×*g* to remove floating cells. Cell lysis and protein extraction was performed with the Pierce™ IP Lysis Buffer (87788, Thermo Fischer Scientific, Waltham, MA, USA). For the ELISA, the cell supernatant was used non-diluted, whereas the cell lysis was diluted 1:2 for the pro-NGF and 1:3 for the pro-BDNF with the Assay Diluent A. ELISA was performed according to the manufacturer’s instructions. Absorbance was measured at 450 nm with the Apollo ELISA Reader - Berthold Technologies.

### Statistics

Statistical analyses were conducted using GraphPad Prism 8. Details regarding biological replicates and statistical analyses are provided in the figure legends. At least three independent sets of experiments were performed for qPCR to ensure reproducibility. RNAseq differential expression analysis was conducted using SARTools. Genes were filtered for low counts using a cpmCutoff of 1, Trimmed Mean of M-values (TMM) was used for normalization and p-values were adjusted using the Benjamini-Hochberg (BH) correction to control the false discovery rate (FDR).

## Results

### High glucose induces DNA damage and cell death in DA neurons

**Fig. 1:**
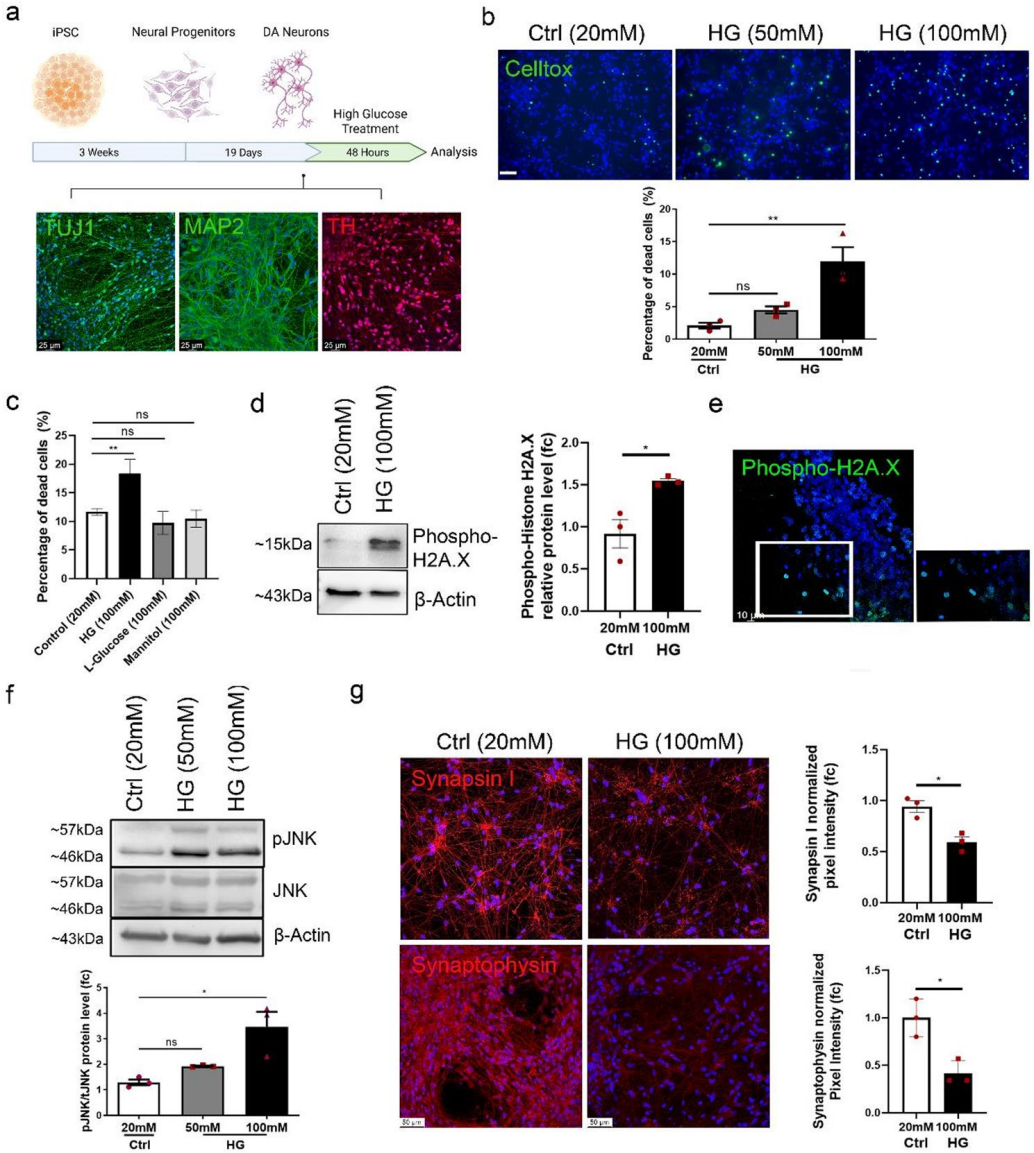
High glucose has a pro-apoptotic effect on iPSC-derived dopaminergic neurons. **a** Schematic of cell differentiation and glucose treatment procedures. Dopaminergic neurons were differentiated from Neural Progenitor Cells (NPCs). At Day 19 of differentiation, neurons were exposed to HG (50mM or 100mM) for 48 h. Cell identity was confirmed with immunostaining for the neuronal markers TUJ1 (green), MAP2 (green) and the dopaminergic marker TH (red). Scale bar 25 μm. **b** Celltox cytotoxicity assay. Representative images and quantification of dead neurons(green) after cell exposure to HG for 48 h. Scale bar 50 μm. **c** CellTox cytotoxicity assay. Graph showing the percentage of dead neurons upon exposure to 100mM d-glucose (HG), 100mM l-Glucose or 100mM Mannitol for 48 h. **d** Western blot analysis and quantification of the DNA damage marker Phospho-Histone H2A.X (Ser139) in Control and HG-treated neurons. Phospho-Histone H2A.X levels were normalized to β-Actin. **e** Immunofluorescence staining showing neurons positive for Phospho-Histone H2A.X (Ser139) (green) after HG treatment for 48 h. Scale bar 10 μm. **f** Western Blot for phospho-JNK and JNK in Control and HG-treated neurons. Graph showing the ratio of pJNK/JNK/β-Actin. **g** Immunofluorescence analysis of Synapsin I and Synaptophysin in Control and HG- treated neurons. Scale Bar 50 μm. Data are presented as mean ± SEM from three biological replicates derived from three independent iPSC lines. For **b**, **c** and **f** statistical significance was assessed using one-way ANOVA with Turkey’s multiple comparisons test (*P < 0.05; **P < 0.01). For d statistical significance was assesed with unpaired t-test (*P < 0.05). For g the statistical significance was assessed using unpaired t test (*P < 0.05). Full-length blots are presented in Figure S4

We first generated neural progenitor cells (NPCs) from three human iPSC lines using small molecules as previously described [73]. NPCs were then differentiated towards DA neurons. The resulting cell population was characterized by immunostaining for the neuronal markers TUJ1 and MAP2 and the dopaminergic marker TH (Fig. 1a). Neurons were differentiated and maintained in basal medium containing 20 mM d-glucose. Upon achieving terminal differentiation, neurons were exposed to higher glucose (HG) concentrations by supplementing the medium with 50 mM or 100 mM d-glucose for 48 h. Glucose levels that exceed physiological concentrations were necessary due to metabolic adaptation of the cells to elevated glucose in standard culture media, which limited the induction of measurable neurotoxic effects at lower concentrations.

Assessment of cell viability using the CellTox cytotoxicity assay revealed that treatment with 100 mM glucose induced significant neuronal cell death, while a trend toward increased toxicity was also observed at 50 mM glucose. The fraction of dead cells increased from 2.09 ± 0.44% in control cells to 11.9 ± 2.1% following treatment with 100 mM high glucose (HG) (Fig. 1b). To examine whether osmotic stress contributes to high glucose–induced neurotoxicity, neurons were treated with equimolar concentrations of the non-metabolizable l-glucose and mannitol. As shown in Fig. 1c, exposure to 100 mM d-glucose resulted in significant neuronal cell death, whereas equivalent concentrations of l-glucose or mannitol had no effect on neuronal viability indicating that the observed neurotoxicity is driven by glucose metabolism rather than osmotic stress (Fig. 1c). Glucose overload is known to induce oxidative stress and to render cells more vulnerable to DNA damage [30, 72]. Indeed, immunocytochemistry and Western blot analyses of phosphorylated H2AX, a marker of double-stranded DNA breaks, confirmed that DA neurons accumulate DNA damage in response to HG (Fig. 1d, e). In accordance with the apoptotic phenotype, we found elevated phosphorylation levels of the JNK protein in 100mM glucose-treated neurons suggesting activation of the JNK signaling (Fig. 1f). Finally, in agreement with studies reporting deregulation of synaptic proteins and synaptic transmission under hyperglycemia in hippocampal neurons [75, 91], we found that HG treatment significantly decreased the expression of Synapsin I and Synaptophysin in DA neurons implying disturbance of synaptic plasticity (Fig. 1g). Overall, high glucose has a pleiotropic toxic effect on DA neurons eventually leading to cell death.

### Global transcriptomic analysis of high glucose-treated neurons shows induction of stress related processes

**Fig. 2:**
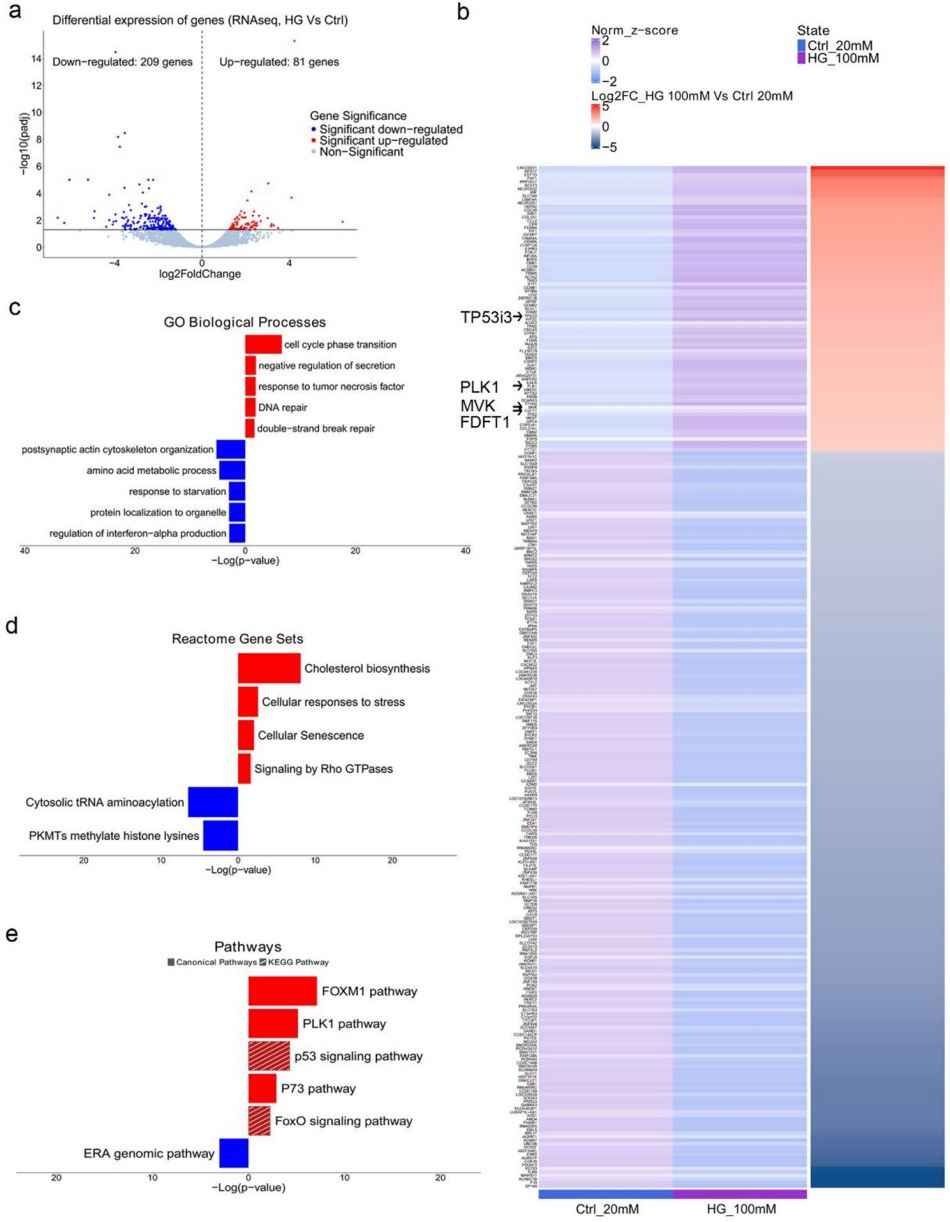
Differential gene expression analysis by RNA-seq of neurons exposed to high glucose. **a** Up- (81; red dots) and down- (209; blue dots) regulated differentially expressed genes (DEGs) (p adj < 0.05) were identified and plotted on a volcano plot to show genes with perturbed expression in HG-treated neurons (100 mM d-glucose, 48 h) compared to Control neurons. **b** Heatmap showing averaged z-scores across replicates and Log2 Fold Change (LFC) for all differentially expressed genes (DEGs) (padj < 0.05) identified between control neurons (blue) and HG-treated (100 mM d-glucose, 48 h) neurons (purple). DEGs are sorted based on descending LFC values. **c**–**e** Functional enrichment analysis using Metascape (Enrichment P Cutoff = 0.05). Bidirectional bar plots showing a selection (see supplementary table for full list and details) of significant terms of GO Biological Processes, Reactome Gene Sets and Pathways, respectively. Both directions show positive values of −Log(p-value) (significance of enrichment). Term bars are color coded based on the gene list of origin. Red bars, enriched terms using all up-regulated genes as input; blue bars, enriched terms using all down-regulated genes as input. Data represent biological replicates derived from independent cell differentiation experiments of one iPSC line

To gain deeper insights into the molecular impact of hyperglycemia on DA neurons, we performed RNA sequencing to compare the global gene expression profiles between HG-treated and Control neurons. Glucose treatment induced transcriptional changes in neurons, with 81 genes up-regulated and 209 genes down-regulated compared to non-treated cells (p adj < 0.05) (Fig. 2a, b Additional File 1: Figure S1, Additional File 2: Table S1, Additional File 3: Table S). Gene Ontology (GO) and Reactome Gene Set (RGS) enrichment analyses of the up-regulated genes indicated induction of processes related to cellular stress including DNA damage repair, negative regulation of secretion and cellular senescence (Fig. 2c, d, Additional File 4: Table S3). Notably, genes involved in cholesterol biosynthesis, such as *MVK* and *FDFT1*, were upregulated, consistent with previous findings linking hyperglycemia to enhanced cholesterol production [27, 82] (Fig. 2b, d). Interestingly, the most significantly enriched GO term was “cell cycle phase transition”, despite DA neurons being post-mitotic. This suggests HG triggers compensatory expression of cell survival and cycle regulatory genes, including *BIRC5* (a component of the chromosomal passenger complex) and *CCNB1/2* (Additional File 3: Table S2, Additional File 4: Table S3, Additional File 6: Table S5). In line with the observed induction of DNA damage in HG-treated neurons, pathway analysis revealed induction of PLK1. PLK1 is part of the DNA damage response (DDR) mechanism that protects cells from entering the cell cycle with damaged DNA [40] (Fig. 2e). Additionally, pro-apoptotic pathways including p53 and p73 were induced (Fig. 2e). In parallel, analysis of the down-regulated genes indicated changes in the post-synaptic cytoskeleton organization and affected ERA genomic pathway that associates with neuroprotection in DA neurons [5, 11] (Fig. 2c, e, Additional File 5: Table S4, Additional File 6: Table S5). Overall, the RNA sequencing analysis corroborated the experimental evidence of DNA damage and cell death induction under HG.

### p75NTR is upregulated and participates in high glucose-driven cell death in DA neurons

Considering the pro-apoptotic potential of p75NTR receptor in different types of neurons in the CNS and its previous involvement in diabetic peripheral neuropathy [20] we set out to investigate its role in high glucose-induced toxicity in dopaminergic neurons.

**Fig. 3:**
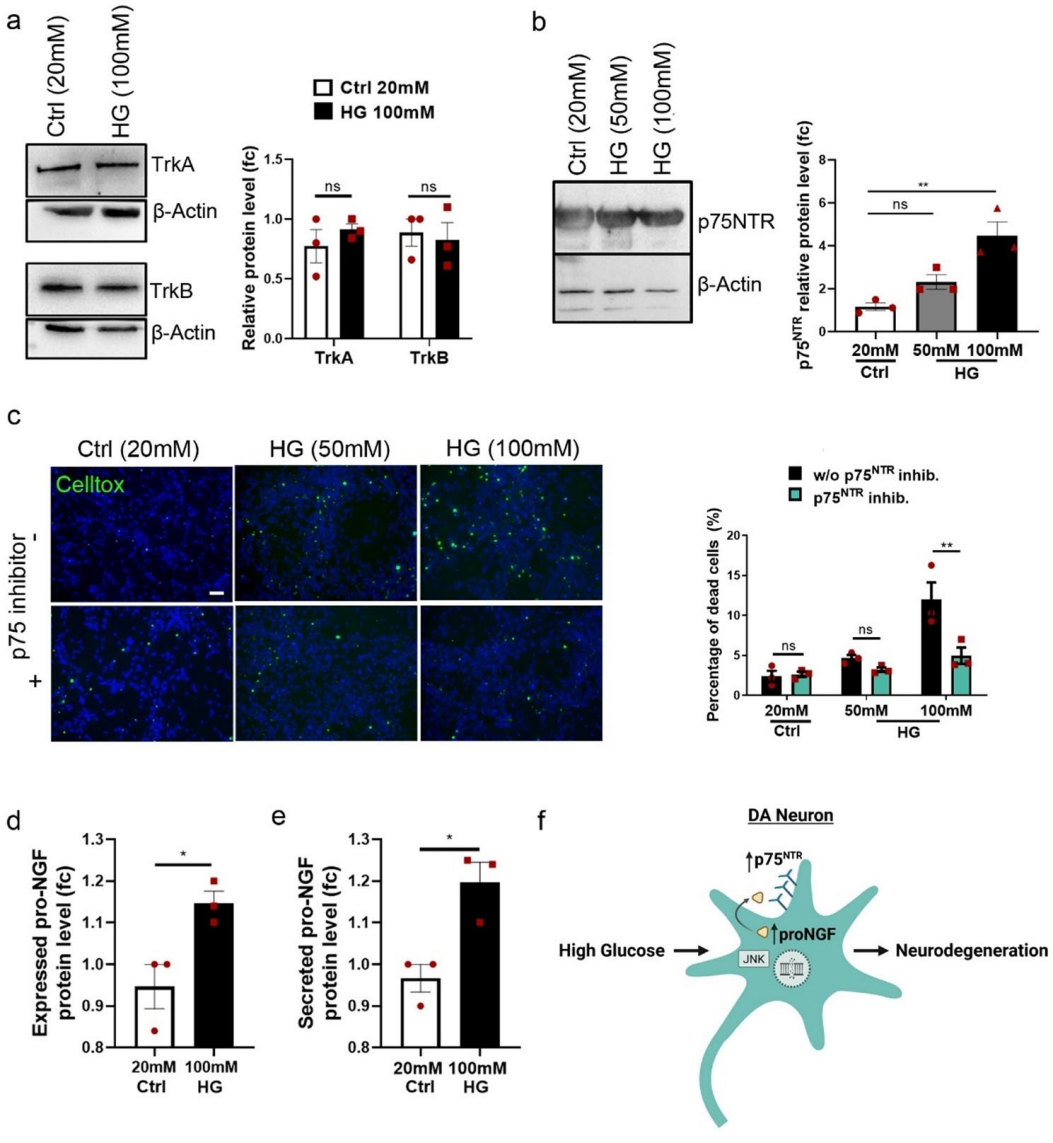
Pro-NGF/p75NTR axis is up-regulated in glucose neurotoxicity in DA neurons. **a** Western blot analysis and quantification of TrkA and TrkB in Control and HG- (100mM) treated neurons for 48 h. Protein levels were normalized to β-Actin. **b** Western blot analysis and quantification of p75NTR in Control and HG (50mM, 100mM) treated neurons for 48 h. Protein levels were normalized to β-Actin. **c** Celltox cytotoxicity assay. Representative photos and quantification of dead (green) neurons after treatment with HG (50mM, 100mM) and/or p75NTR inhibitor (2.5 ng/ml, anti-p75 Receptor antibody (MC-192) Abcam) for 48 h. Scale bar 50 μm. **d**, **e** ELISA quantification of **d** pro-NGF protein levels in the cell lysate and **e** the secreted pro-NGF protein levels in the supernatant of DA neurons treated with HG (100mM) for 48 h. **f** Schematics of the up-regulated pro-NGF/p75NTR axis in DA neurons upon treatment with HG. For **a**–**e** data are presented as mean ± SEM of three biological replicates derived from three independent iPSC lines. For **a** and **c** statistical significance was assessed with a two-way ANOVA and Sidak’s multiple comparisons test (**P < 0.01). For **b** statistical significance was assessed with an ordinary one-way ANOVA and Turkey’s multiple comparisons test (**P < 0.01). For **d** and **e** statistical significance was evaluated with an unpaired t-test (*P < 0.05). Full-length blots are presented in Figure S5

First, we analyzed the expression pattern of neurotrophin receptors. TrkA, TrkB and p75NTR receptors are expressed in human DA neurons (Fig. 3a, b, Additional File 1: Figure S2a). Although the expression of the neuroprotective Trk receptors remains unaffected (Fig. 3a), neuronal treatment with 100mM glucose significantly upregulated the expression of the p75NTR receptor (Fig. 3b). p75NTR has no intrinsic catalytic activity but signals through interactions with effector proteins that engage different signaling pathways [14]. To further investigate the role of p75NTR in glucose neurotoxicity, we used a p75NTR neutralizing antibody (MC-192) that binds the extracellular domain of the receptor serving as a selective inhibitor [45, 65]. Strikingly, inhibition of p75NTR effectively ameliorated the neurotoxic effect of high glucose indicating that p75NTR mediates glucose neurotoxicity in DA neurons. Inhibition of p75NTR activity decreased the number of dead cells from 11.96% ± 2.1% to 4.9% ±1.02% in 100mM HG (Fig. 3c). The protective effect of p75NTR inhibition observed in the CellTox cytotoxicity assay was also confirmed by using TUNEL assay, which specifically detects apoptotic cells (Additional File 1: Figure S2b).

p75NTR can differentially regulate neuronal survival in a ligand- and context-dependent manner. It promotes neuronal survival when bound to mature neurotrophins in the presence of pro-survival Trk receptors, whereas it can induce pro-apoptotic signaling when bound to pro-neurotrophins [56]. Therefore, we next evaluated the expression and secretion levels of pro-apoptotic pro-neurotrophins, the pro-NGF and pro-BDNF, in DA neurons. ELISA measurement of pro-NGF showed a significant increase in the synthesis (Fig. 3d) and secretion (Fig. 3e) of pro-NGF in DA neurons in response to HG. In contrast, the levels of pro-BDNF remained unaffected (Additional File 1: Figure S2c, d).

To further confirm the hypothesis that the pro-NGF/p75NTR axis participates in glucose-induced neurotoxicity, we pharmacologically targeted p75NTR and its coreceptor Sortilin, a key component of the pro-NGF signaling complex. Treatment with 400nM LM11A-31, a small-molecule p75NTR modulator that disrupts pro-NGF–p75NTR pro-apoptotic signaling [25, 55, 83], significantly reduced high-glucose–induced neuronal death (Additional File 1: Figure S2e). Likewise, inhibition of Sortilin with the selective antagonist AF38469 attenuated neuronal loss under hyperglycemic conditions (Additional File 1: Figure S2e). Collectively, our data suggest the activation of an autocrine pro-apoptotic pro-NGF/p75NTR signaling in DA neurons, when exposed to high glucose (Fig. 3f) and demonstrate that modulation of p75NTR activity can alleviate glucose neurotoxicity.

### High glucose increases neuronal vulnerability to 6-OHDA toxicity, an effect mitigated by p75NTR inhibition

**Fig. 4:**
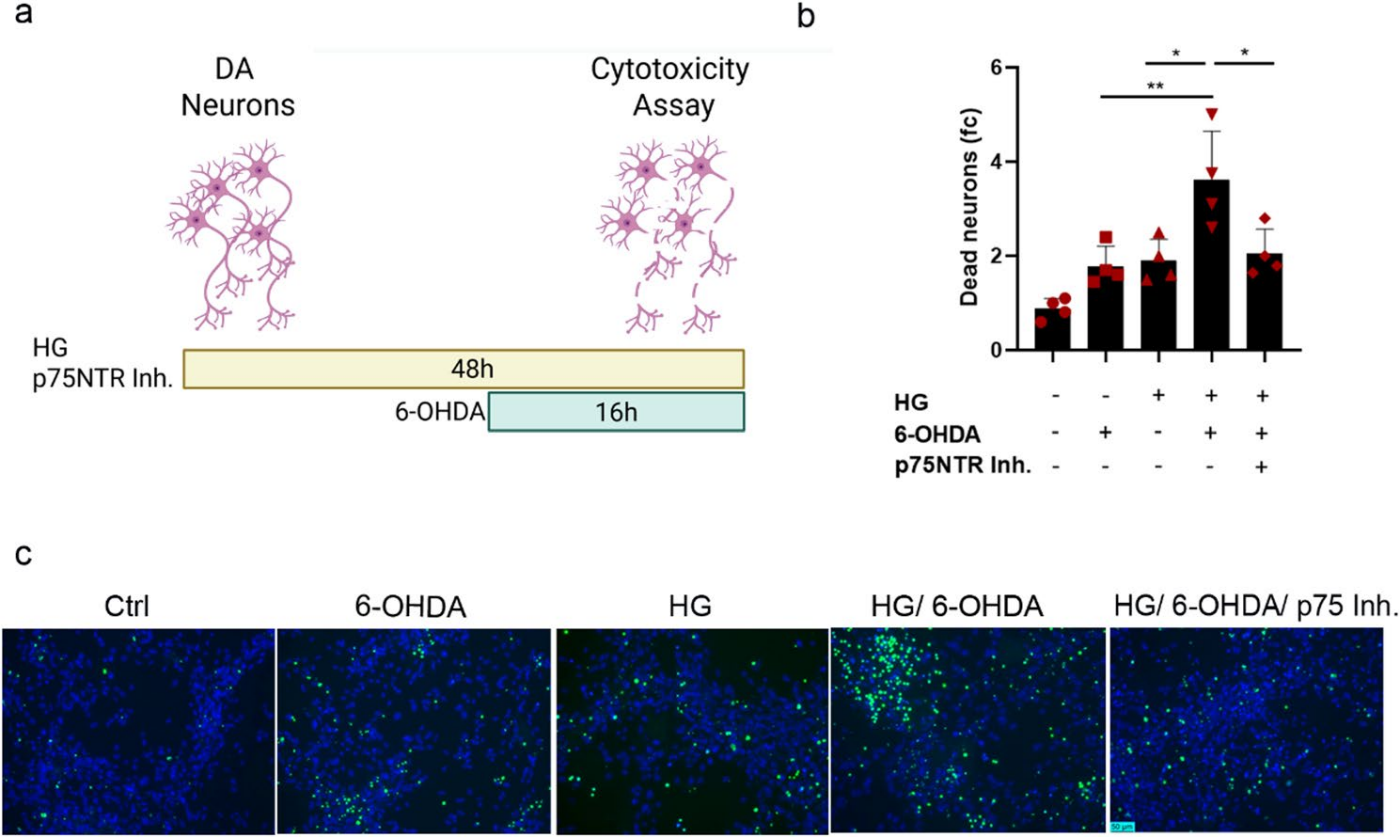
p75NTR inhibition attenuates the high glucose- induced sensitization to 6-OHDA toxicity. Celltox cytotoxicity assay. **a** Schematic overview of the experimental design. **b** Quantification and **c** representative images of dead neurons (green) upon exposure to HG (100mM) and p75NTR inhibitor (2.5 ng/ml, anti-p75 Receptor antibody (MC-192) Abcam) for 48 h and 6-OHDA (50 µM) during the last 16 h. Scale bar 50 μm. For b data are shown as mean ± SEM of four biological replicates derived from three independent iPSC lines. Statistical significance was evaluated with ordinary one-way ANOVA and Turkey’s multiple comparisons test (*P < 0.05; **P < 0.01)

Considering the increased risk of diabetic patients to develop dopaminergic dysfunction and PD, we next sought to determine whether high glucose increases DA neuron vulnerability to well established exogenous neurotoxic stimuli. We first treated neurons with the neurotoxin 6-hydroxydopamine (6-OHDA), which induces oxidative stress and impairs mitochondrial activity selectively in DA neurons [42, 49]. Neurons were exposed to HG for 48 h with or without 50 µM 6-OHDA co-administered during the last 16 h (Fig. 4a). Although 6-OHDA induced only a mild, non-significant toxicity in dopaminergic neurons in control glucose conditions, likely due to the short incubation time, it caused significant cell death under hyperglycemic conditions (Fig. 4b, c). Importantly, inhibition of p75NTR activity significantly reduced the neurotoxic effect of 6-OHDA in HG-treated neurons suggesting its neuroprotective potential in a PD relevant model (Fig. 4b, c).

Next, we challenged DA neurons with Amyloid-β oligomers. Amyloid-β burden has been described in PD patients and has been associated with cognitive impairment in PD [31, 57]. Additionally, although DA neurons are not the primarily affected neuronal cell population in Alzheimer’s Disease (AD), preclinical and neuropathological evidence associates loss of DA neurons and low levels of dopamine with memory deficits in AD [62]. Therefore, we sought to determine the impact of high glucose on DA neuron susceptibility to the neurotoxic effect of Amyloid-β 1–42 oligomers, which are major components of amyloid plaques [34]. We treated DA neurons with HG and 10µΜ Amyloid-β (1–42) oligomers for 48 h. Amyloid-β (1–42) did not affect the survival of dopaminergic neurons under control glycemic conditions, however high glucose sensitized neurons to Amyloid-β-induced toxicity (Additional File 1. Figure S3 a, b). These findings underline the influence of hyperglycemic events on the progression of neurodegeneration in PD patients with Amyloid-β burden and suggest an interlink between DM, PD and cognitive dysfunction.

### BNN27 exerts a neuroprotective effect in DA neurons via p75NTR and TrkA receptors

**Fig. 5:**
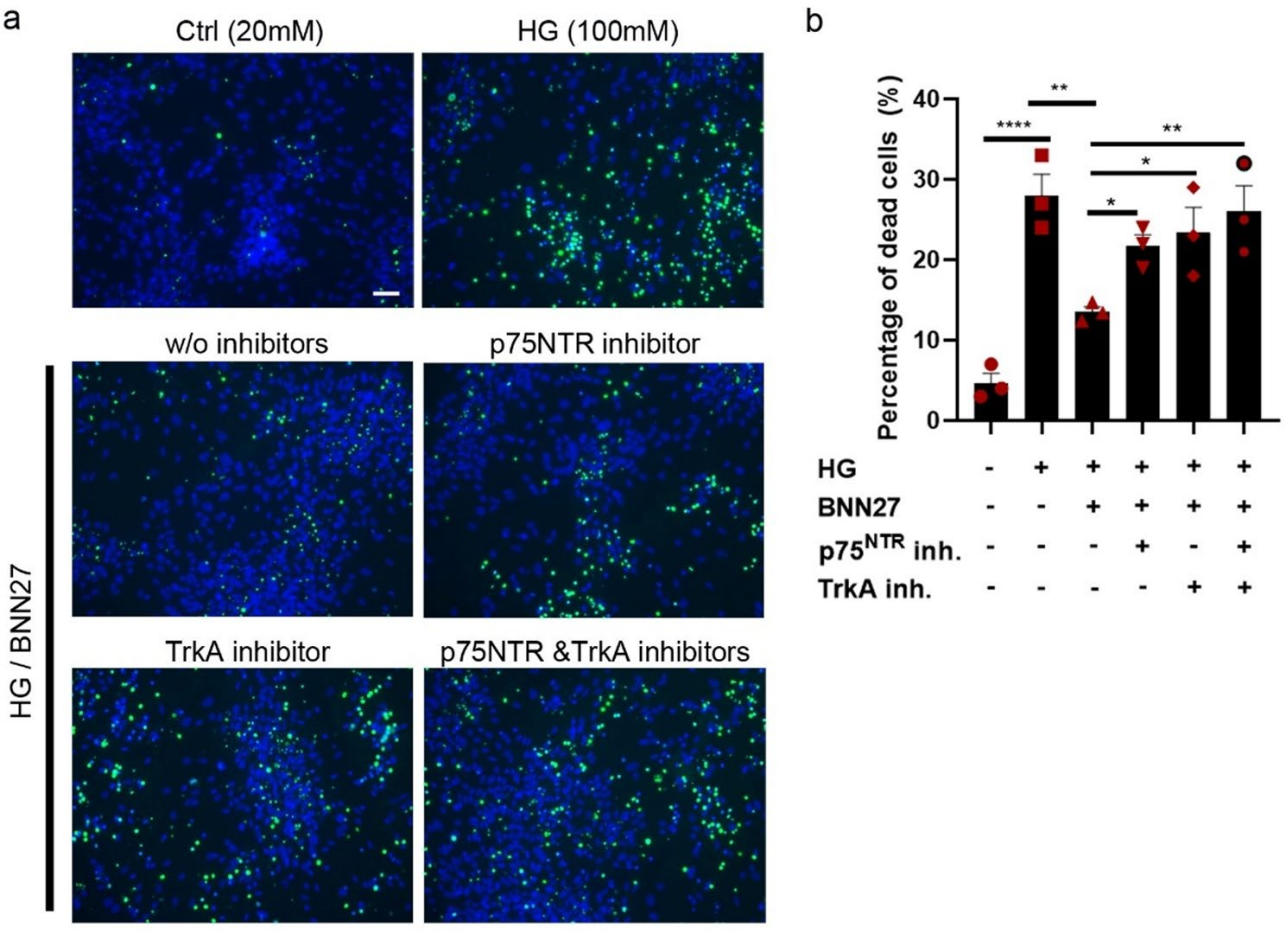
BNN27 mitigates the neurotoxic effect of high glucose via p75NTR and TrkA receptors. Celltox cytotoxicity assay. **a** Representative images and **b** quantification of dead neurons (green) upon treatment with HG (100mM), BNN27 (1 µM), p75NTR inhibitor (2.5 ng/ml, anti-p75 Receptor antibody (MC-192) Abcam) and/or TrkA inhibitor (20 µM, GW441756, G-190, Alomone labs, Jerusalem, Israel) for 48 h. Scale bar 50 μm. Data are shown as mean ± SEM of three biological replicates derived from three independent iPSC lines. Statistical significance was evaluated using ordinary a one-way ANOVA and multiple comparisons test versus the indicated condition (*P < 0.05, **P < 0.01; **** *P* < 0.0001)

Based on the pro-apoptotic function of p75NTR, we next questioned whether neurotrophin signaling could be therapeutically targeted to alleviate HG-driven neurodegeneration. We tested a synthetic small-sized 17-spiro-steroid analog that acts as NGF mimetic, BNN27, which has gained interest as a therapeutic molecule for neuroprotection in diabetic retinopathy [41] and Alzheimer’s Disease [45]. Previous publications of our and other groups have shown that BNN27 acts as a selective activator of both NGR receptors, TrkA and p75NTR receptors, promoting the survival of multiple types of neurons [45, 66]. Exposure of dopaminergic neurons to 1 µM BNN27 significantly alleviated high glucose-induced cell death. This protective effect was abolished by inhibition of p75NTR or TrkA, either alone or in combination indicating that BNN27 exerts its protective effects through both receptors (Fig. 5a, b). Collectively, our findings highlight BNN27 as a promising neurotrophin-based therapeutic candidate capable of mitigating HG- induced dopaminergic neurodegeneration.

### High glucose enhances the inflammatory potential of astrocytes and induces the secretion of neurotoxic factors

**Fig. 6:**
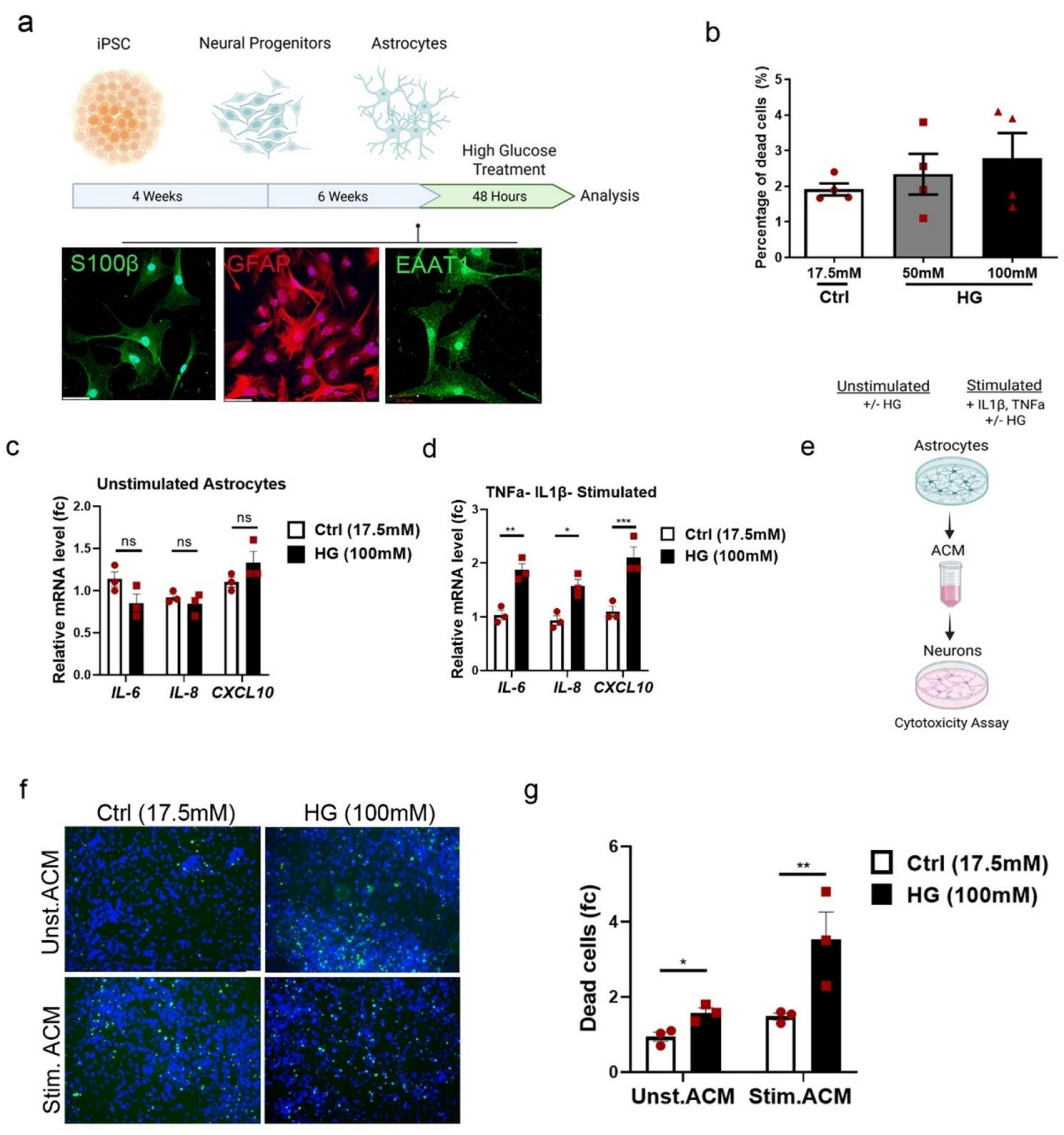
High glucose amplifies the pro-inflammatory response of astrocytes and indirectly contributes to neurodegeneration. **a** Schematic of astrocyte differentiation and glucose treatment procedures. Astrocytes were differentiated and treated with HG for 48 h prior to analysis. Representative photos showing S100β+, GFAP + and EAAT1 + astrocytes on differentiation day 50. Scale bar 50 μm. **b** Celltox cytotoxicity assay. Quantification of dead astrocytes upon treatment with HG (50mM and 100mM). **c**, **d** mRNA levels of *IL-6*,* IL-8* and *Cxcl10* in astrocytes treated with **c** HG (100mM) in unstimulated conditions (Unstimulated) and **d** HG (100mM) stimulated with TNFa (30 ng/ml) and IL-1β (10 ng/ml) for 48 h (TNFa- IL1β- stimulated). Gene expression was normalized to *Beta-Actin*. **e** Schematic of Astrocyte Conditioned Medium (ACM) collection and its application to neuronal cultures for 48 h. **f** Celltox cytotoxicity assay in neurons treated with ACM from un- and stimulated astrocytes. +/− HG for 48 h. Representative photos and quantification of dead neurons (green). Scale bar 50 μm. For **b**, data are shown as mean ± SEM of four biological replicates. Statistical significance was evaluated with one-way ANOVA (*P* = 0.5). For **c**, **d**, and **g**, data are shown as mean ± SEM of three biological replicates derived from three independent iPSC lines. Statistical significance was evaluated with two-way ANOVA and Sidak’s multiple comparisons test (*P < 0.05; **P < 0.01)

The current consensus in the literature is that neuroinflammation is a key interlink between DM and neurodegenerative diseases [54]. Astrocytes are primary homeostatic cells in the brain but in response to stress stimuli, they display phenotypic changes that may increase the risk of neurodegeneration [12]. In this context, we investigated the involvement of human astrocytes on dopaminergic neurodegeneration in hyperglycemic condition. We differentiated human iPSCs towards astrocytes as previously described [69]. Cell identity was characterized via immunostaining for the astrocyte markers GFAP, S100β and EAAT1 (Fig. 6a). Mature astrocytes were treated with HG (100mM d-glucose) for 48 h to simulate hyperglycemia. Analysis of cell survival showed no apoptotic effect of HG on astrocytes indicating that astrocytes are more resistant to HG compared to neurons (Fig. 6b). To explore the influence of HG on astrocyte activation, we analyzed the expression of inflammatory markers in the presence or absence of exogenous pro-inflammatory stimuli. Astrocytes were treated with HG and/or 30 ng/ml TNFa and 10 ng/ml IL-1β to induce activation. qPCR analysis showed increased gene expression of *IL-6*,* IL-8* and *Cxcl10* in HG-treated stimulated but not in unstimulated astrocytes (Fig. 6c, d) implying that HG was not adequate to activate these genes but exacerbates astrocyte responsiveness to pro-inflammatory stimuli. Finally, to evaluate how HG-treated astrocytes interfere with neuronal survival, we collected the Astrocyte Conditioned Medium (ACM) of both cytokine-stimulated and unstimulated astrocytes. DA neurons were exposed to ACM for 48 h without additional glucose (Fig. 6e). Notably, ACM from hyperglycemic astrocytes—both with and without exogenous cytokine stimulation—caused significant neuronal death, with a stronger effect observed in the cytokine-stimulated group. Specifically, ACM from HG-treated astrocytes increased neuronal death by 1.6-fold compared to the control condition, while ACM from cytokine- and HG-treated astrocytes led to a 2.3-fold increase (Fig. 6f, g). Although cytokine expression levels were unchanged in non-stimulated cells, other secreted factors with toxic properties may account for this effect. Conclusively, our results indicate that HG enhances the inflammatory potential of astrocytes and induces the secretion of neurotoxic factors with a harmful effect on DA neurons.

### High glucose leads to neurotoxic microglia response

**Fig. 7:**
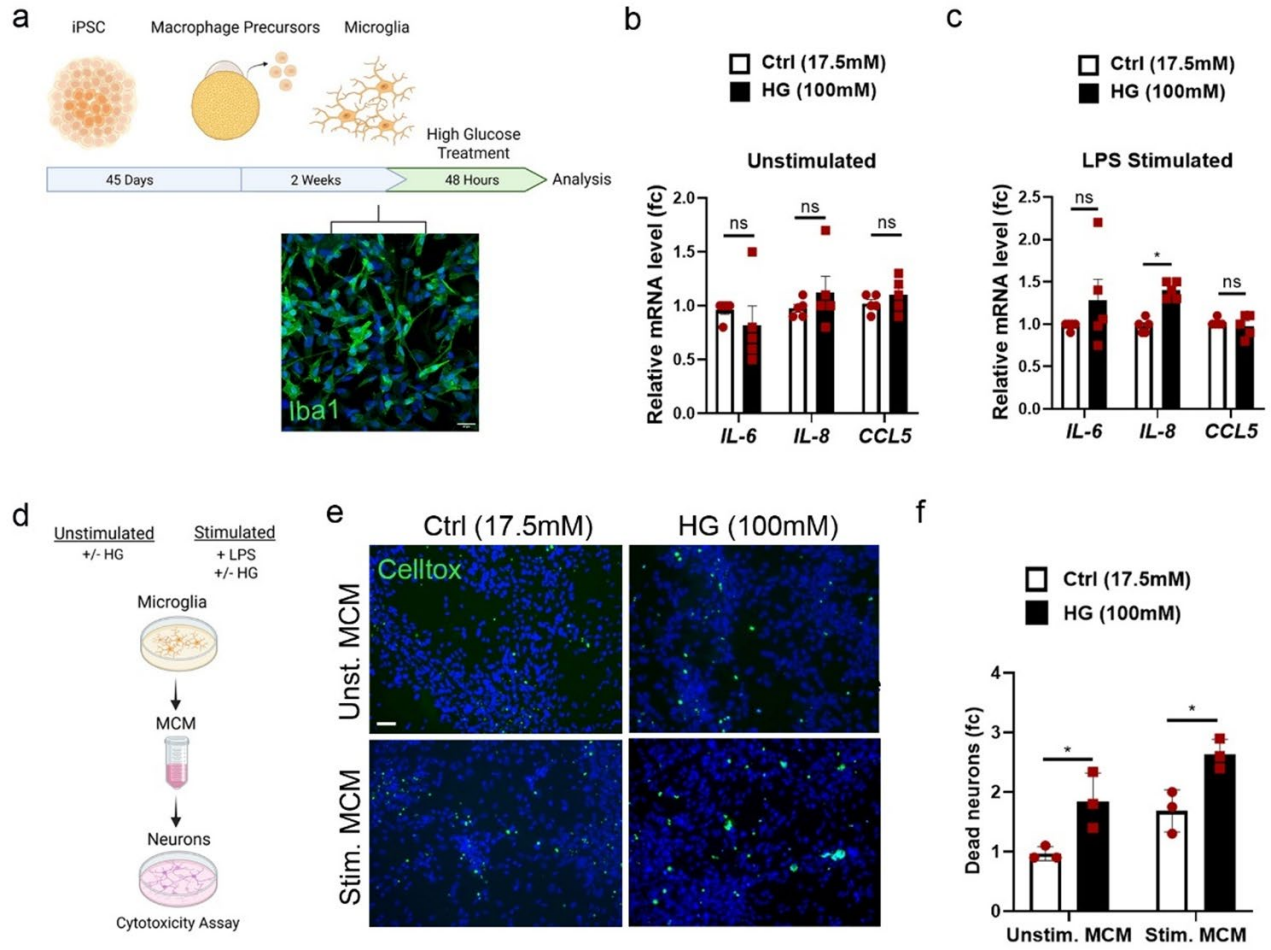
High glucose induces the release of neurotoxic factors by microglia. **a** Schematic of iPSC differentiation to Iba1 + microglia (green) and glucose treatment. Microglia were treated with high glucose (100mM) for 48 h. Scale bar 20 μm. **b**, **c** mRNA levels of *IL-6*,* IL-8*, and *Ccl5* in **b** microglia treated with HG (100 mM) in unstimulated conditions (Unstimulated), and **c** microglia treated with HG in LPS (100 ng/mL) stimulated conditions (LPS-stimulated). Gene expression levels were normalized to *Beta-Actin*. **d** Schematic of Microglia Conditioned Medium (MCM) collection. DA neurons were treated with 1:1 MCM/Neuronal differentiation medium for 48 h. **e**, **f** Celltox cytotoxicity assay in neurons treated with MCM. **e** Representative photos and **f** quantification of dead neurons upon treatment with MCM collected from unstimulated and LPS-stimulated microglia for 48 h. Scale bar 50 μm. For **b** and **c**, data are shown as mean ± SEM of five biological replicates. Statistical significance was evaluated with a two-way ANOVA and Sidak’s multiple comparisons test (*P < 0.05). For **f**, data are shown as mean ± SEM of three biological replicates and statistical significance was evaluated with a two-way ANOVA and Sidak’s multiple comparisons test (*P < 0.05). Biological replicates represent independent differentiation experiments from a single iPSC line

Microglia, the resident macrophages of the CNS, are the main mediators of neuroinflammation in the brain. Activated microglia may impair neuronal activity through the release of cytokines, chemokines and glutamate [29]. To explore the impact of high glucose on human microglia and the secondary effect on DA neuropathology, we generated Iba1 + microglia from human iPSCs as previously described [33] (Fig. 7a). Microglia were exposed to 100mM d-glucose for 48 h and/or 100 ng/ml LPS during the last 6 h to simulate a pro-inflammatory microenvironment. Alike astrocytes, qPCR analysis for *IL-6*,* IL-8* and *Ccl5* showed that microglia exposure to HG did not induce the expression of these inflammatory markers (Fig. 7b) though LPS stimulation enhanced the expression of *IL-8* under HG (Fig. 7c). We next collected the microglia-conditioned media (MCM) for neuronal treatment (Fig. 7d). The MCM from HG-treated microglia, both un- and LPS-stimulated, increased neuronal cell death. The fraction of dead neurons was increased 1.9-fold and 1.5-fold after cell exposure to MCM from hyperglycemic un-stimulated and LPS-stimulated microglia, respectively, compared to Control (Fig. 7e, f). In summary, our data demonstrate that elevated glucose levels trigger microglia to secrete factors that impair DA neuron viability, a phenotype also observed in astrocytes. Our results point to an important role of glial cells in the neurological complications of diabetes and highlight neuron-glia crosstalk as a target for intervention in future therapeutic avenues.

## Discussion

Hyperglycemia is a hallmark of DM but transient hyperglycemic events may arise also in other conditions, such as post Traumatic Brain Injury [71], stress-hyperglycemia in patients with acute illness [24] or gestational hyperglycemia [76]. Prospective cohort studies support that Type 2 DM increases the risk for PD and accelerates PD progression [17, 21, 23, 47, 68], while antidiabetic agents are being evaluated as modifiers of PD pathophysiology [2, 88]. However, the underlying mechanisms of hyperglycemia-induced dopaminergic dysfunction remain poorly understood. To date, most evidence has been derived from animal models, limiting the translational relevance and clinical interpretation of the findings. Our study assessed the impact of high glucose on human iPSC-derived dopaminergic neurons and investigated their crosstalk with glial cells in order to better understand the pathological manifestations of acute hyperglycemia in the brain in an in vitro human-relevant model.

In this study, iPSC-derived neurons, astrocytes, and microglia were exposed to 100 mM glucose for 48 h to model acute hyperglycemic stress. These glucose levels exceed physiological concentrations (blood: ~5.5 mM normoglycemia, up to ~ 20 mM in hyperglycemia; brain: ~1 mM normoglycemia, 5 mM in hyperglycemia) [39]. However, iPSCs and their derived cells are routinely maintained in media with higher basal glucose (17.5–20 mM), which may induce metabolic adaptations during prolonged in vitro culture. Consequently, higher glucose concentrations were required to reliably elicit acute stress and perform mechanistic studies under controlled conditions. The 48 h treatment period aligns with previous studies modeling acute hyperglycemia in vitro [53, 64].

STZ-treated rodents exhibit decreased dopamine levels and dopaminergic neurodegeneration [68, 74]. Accordingly, our analysis showed that exposure of human DA neuron to HG (100mM) induces DNA damage, changes in synaptic protein expression and eventually cell death. Additionally, HG induces DA neuron susceptibility to 6-OHDA and Amyloid-β-driven cytotoxicity emphasizing the importance of glucose regulation in patients with PD or high Amyloid-β burden due to aging. Transcriptomic analysis confirmed the induction of DNA damage response and cell death-related pathways, like p53, in neurons when exposed to HG. However, the use of a single iPSC line for the RNA-seq analysis represents a limitation in generalizing these findings across genetically diverse backgrounds. Overall, our model recapitulates many glucotoxicity features described in diabetic animal models and can serve as a platform to identify mediators of glucose neurotoxicity in the human CNS.

Impaired neurotrophin signaling has previously been correlated with brain damage in diabetic patients. T2DM patients have lower serum BDNF [36], while up-regulation of BDNF reduces neuroinflammation [35] and protects hippocampal neurons from hyperglycemia-driven apoptosis [91]. Furthermore, pro-NGF is up-regulated in the retina of STZ-treated rodents and promotes neuroinflammation [61]. Our study implicates for the first time the pan-neurotrophin receptor p75NTR in high glucose-induced neuronal loss in the brain. p75NTR is a transmembrane receptor that is up-regulated in the adult brain in response to injury and disease [3, 80]. p75NTR can promote neuronal survival in conjunction with TrkA when bound to mature neurotrophins [28], whereas the p75NTR-sortilin complex can induce apoptosis upon binding to pro-neurotrophins [79]. Notably, p75NTR signaling has been already implicated in DA neuronal loss in the rotenone model of PD (L. W. Chen et al., [16, 18] as well as in the neurological complications of DM in the PNS and retina [6, 20, 26, 38, 61, 78]. Here, we extend these findings by demonstrating its involvement in hyperglycemia-induced brain pathology. We show that p75NTR is up-regulated and displays a pro-apoptotic function in DA neurons in hyperglycemic conditions. HG treatment up-regulates p75NTR and its pro-apoptotic ligand pro-NGF eventually inducing an autocrine induction of the pro-NGF/p75NTR axis in DA neurons. Importantly, inhibition of p75NTR, either with a neutralizing antibody targeting its extracellular domain or with LM11A-31 which antagonizes pro-NGF-induced neurodegeneration, protected neurons from high glucose-induced cell death. A similar protective effect was observed when Sortilin, a co-receptor of p75NTR involved in pro-neurotrophin/p75NTR-mediated apoptotic signaling, was inhibited [79]. supporting a key role for the pro-NGF/p75NTR pathway in hyperglycemia-mediated neurotoxicity and highlighting the therapeutic potential of p75NTR-modulatory strategies. Further analysis of pro-NGF and p75NTR levels in the CSF of diabetic patients is necessary to confirm their prognostic value in diabetic neurodegeneration.

Although the neuroprotective and anti-inflammatory properties of neurotrophins are well documented, they face therapeutic limitations due to low stability and poor BBB permeability. These challenges have been addressed by developing small synthetic DHEA-derived microneurotrophins, which mimic neurotrophin activity by targeting their receptors and show promise in neuroprotection and regeneration [13, 32]. In this context, we tested the neuroprotective effect of an NGF synthetic mimetic, BNN27, against glucose neurotoxicity in DA neurons. BNN27 is a well characterized microneurotrophin able to penetrate the BBB [8, 43, 85] that has gained interest as a neuroprotective and anti-inflammatory agent in the nervous system acting via both TrkA and p75NTR receptors [41, 45, 66]. Importantly, BNN27 has already been tested successfully against diabetic retinopathy acting as a neuroprotective and anti-inflammatory agent [41]. Here, we demonstrated that BNN27 can alleviate glucose neurotoxicity in DA neurons acting via both p75NTR and TrkA receptors. BNN27-mediated neuroprotective actions have been shown to be dependent on the extracellular domain of p75NTR [67]. We here observed that blocking the extracellular domain of p75NTR using a specific antibody significantly reduced high glucose-induced neurotoxicity. In addition, we found increased expression and secretion of pro-NGF in DA neurons under hyperglycemic conditions. We therefore propose that BNN27 confers neuroprotection, at least in part, by engaging the extracellular domain of p75NTR and preventing its activation by autocrine pro-NGF. Additionally, BNN27 may alter the receptor’s conformation or signaling capacity, shifting its activity away from apoptotic pathways toward a survival-promoting profile, inducing its ability to interact with the pro-survival TrkA receptor. Our results suggest that BNN27, which is currently under pre-clinical investigation against diabetic retinopathy, could be a lead molecule for further evaluation against cases of diabetic encephalopathy.

Accumulating evidence indicates that hyperglycemia-induced alterations in glial cells play a critical role in diabetes-induced neuropathology [50, 90]. To explore changes in neuron-glia interaction in hyperglycemic conditions, we first examined the effect of HG on human iPSC-derived astrocytes and microglia. Our in vitro data indicate that, unlike neurons, human astrocyte survival is not affected by HG, likely due to their higher glycogen storage capacity [81] and stronger antioxidant defenses [19]. Importantly, astrocyte exposure to HG significantly augmented the expression of pro-inflammatory mediators in the presence of inflammatory stimuli. Our results agree with findings from Bahniwal and colleagues who showed that HG induces the secretion of IL-6 and IL-8 in a human astrocytic cell line stimulated with IFNγ and IL1β but not in non-stimulated cells [4]. Similarly, HG treatment upregulated the expression of *IL-8* in LPS-stimulated microglia suggesting that HG sensitizes glial cells to inflammatory stimuli and enhances their reactive response. The absence of a pronounced inflammatory response to HG in our human cell models—unlike the robust activation observed in animal studies in vivo and in vitro [50, 52, 90]—suggests that glial cells possess substantial glucose-buffering capacity, which is compromised under inflammatory conditions. Notably, while HG alone did not significantly upregulate classical pro-inflammatory markers in astrocytes or microglia, conditioned medium from HG-treated glial cells induced apoptosis in dopaminergic neurons. We propose that, under hyperglycemic conditions, glial cells may release neurotoxic factors independent of canonical inflammatory cytokines, like glutamate, nitric oxide and reactive oxygen species [1, 70], or that transient or post-transcriptionally regulated inflammatory signals were not captured at the time of analysis. The neurotoxic effects of the conditioned media were further enhanced when astrocytes and microglia were co-stimulated with additional inflammatory cues. Proteomic and metabolic characterization of the HG-treated glia will unravel the nature of the released molecules and will provide insights into the pathological alterations that contribute to neurodegeneration.

## Conclusions

This study demonstrates that high glucose compromises human dopaminergic neuron survival via activation of the pro-NGF/p75NTR signaling and by triggering indirect, glia-mediated neurotoxic mechanisms. Our findings indicate that targeting p75NTR activity could alleviate dopaminergic neurodegeneration in patients experiencing hyperglycemic episodes and suggest the beneficial effect of the small molecule BNN27 against glucose neurotoxicity. Our research highlights the contribution of glia in neuronal loss in hyperglycemic conditions, suggesting the need for systematic neuroprotective and anti-inflammatory therapeutic strategies. Human iPSC-derived cellular models can be utilized as a platform for elucidating glucose-mediated toxicity parameters and evaluation of anti-diabetic drugs against dopaminergic neuropathology.

## Supplementary Information

Below is the link to the electronic supplementary material.

Supplementary Material 1.Figure S1. Heatmap showing the expression profile of all differentially expressed genes (p adj < 0.05), identified between HG-treated neurons and control neurons. Figure S2. Analysis of p75NTR role in high glucose-treated DA neurons. Figure S3. High glucose triggers DA neuron susceptibility to Amyloid-β 1–42. Figure S4. Uncropped Western Blot images of Fig. 1. Figure S5. Uncropped Western Blot images of Fig. 3.

Supplementary Material 2. Table S1. Annotation of Heatmap rows.

Supplementary Material 3. Table S2. List of DEGs (p adj < 0.05).

Supplementary Material 4. Table S3. Metascape enrichment analysis of the up-regulated genes from the DEG list.

Supplementary Material 5. Table S4. Metascape enrichment analysis of the down-regulated genes from the DEG list.

Supplementary Material 6. Table S5. Gene lists for selected metascape annotations.

## Data Availability

The datasets supporting the conclusions of this article are available in the GEO repository, GSE291145 https://www.ncbi.nlm.nih.gov/geo/query/acc.cgi?acc=GSE291145. The following secure token has been created to allow review of record GSE291145 while it remains in private status: **gzqjakwgrlgvlcr.**.

## Abbreviations

AD: Alzheimer’s disease
AGEs: Advanced glycation end products
ACM: Astrocyte conditioned medium
BBB: Blood brain barrier
CNS: Central nervous system
CSF: Cerebrospinal fluid
DA: Dopaminergic
DDR: DNA damage response
DM: Diabetes mellitus
HG: Hyperglycemia/high glucose
iPSC: Induced pluripotent stem cell
MCM: Microglia conditioned medium
MEF: Mouse embryonic fibroblast
NPC: Neural progenitor cell
PD: Parkinson’s disease
PNS: Peripheral nervous system
P75NTR: p75 neurotrophin receptor
T2DM: Type 2 diabetes mellitus

## Declarations

### Use of Artificial Intelligence

The authors declare that they have not use AI-generated work in this manuscript.
