# Supplementary material for: Targeting p75NTR activity alleviates the neurotoxic effect of high glucose on iPSC-derived dopaminergic neurons
Source: Stem Cell Res Ther. 2026 Mar 21;17:161. doi: 10.1186/s13287-026-04965-y (PMC13126899; doi:10.1186/s13287-026-04965-y)
Supplement: Supplementary file 1 — Supplementary Material 1.Figure S1. Heatmap showing the expression profile of all differentially expressed genes (p adj < 0.05), identified between HG-treated neurons and control neurons. Figure S2. Analysis of p75NTR role in high glucose-treated DA neurons. Figure S3. High glucose triggers DA neuron susceptibility to Amyloid-β 1–42. Figure S4. Uncropped Western Blot images of Fig. 1. Figure S5. Uncropped Western Blot images of Fig. 3. [file 13287_2026_4965_MOESM1_ESM.pdf]

Figure S1

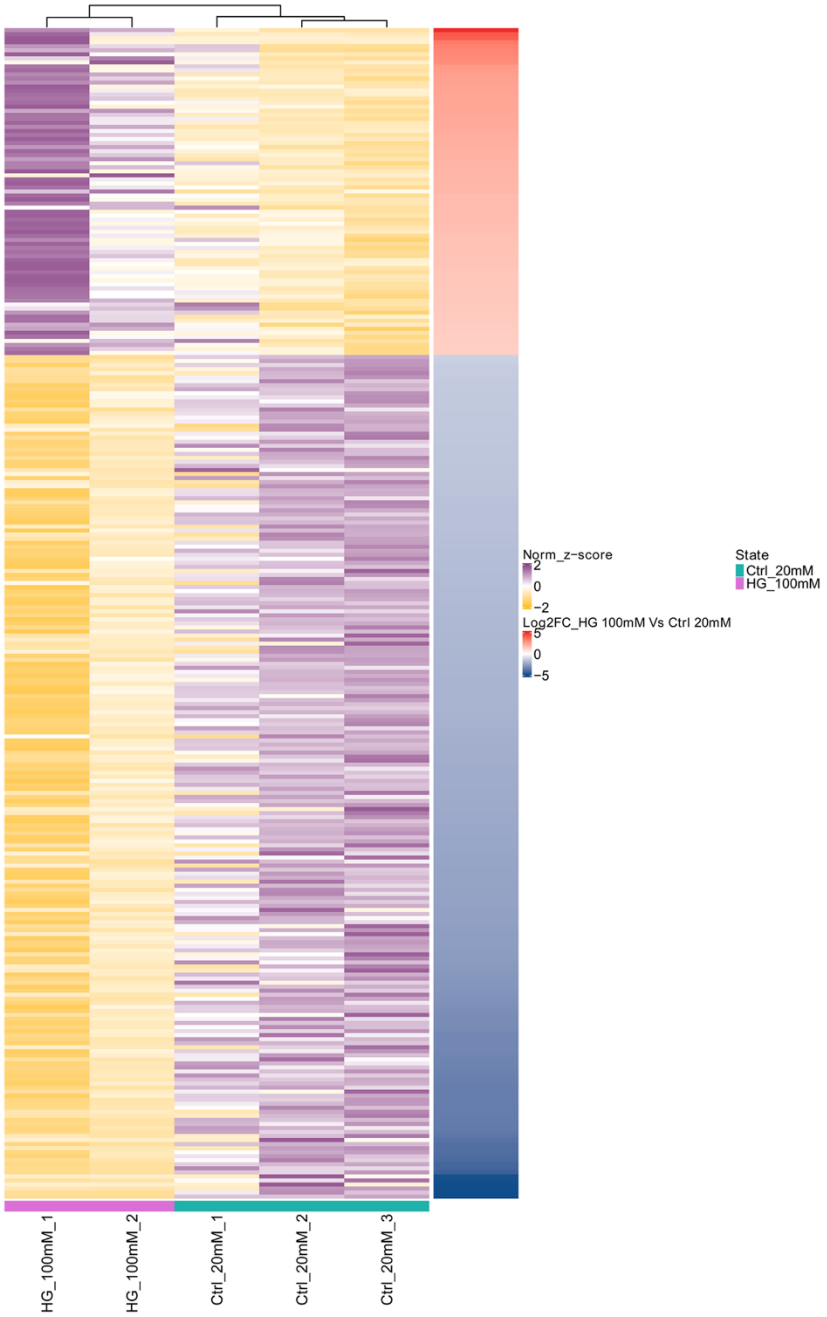

Figure S1. Heatmap showing the expression profile of all differentially expressed genes (p adj < 0.05), identified between HG-treated (100mM D-glucose, 48h) neurons (purple) and control neurons (green). 81 genes were significantly up-regulated and 209 were down-regulated in HG-treated neurons compared to control.

Figure S2

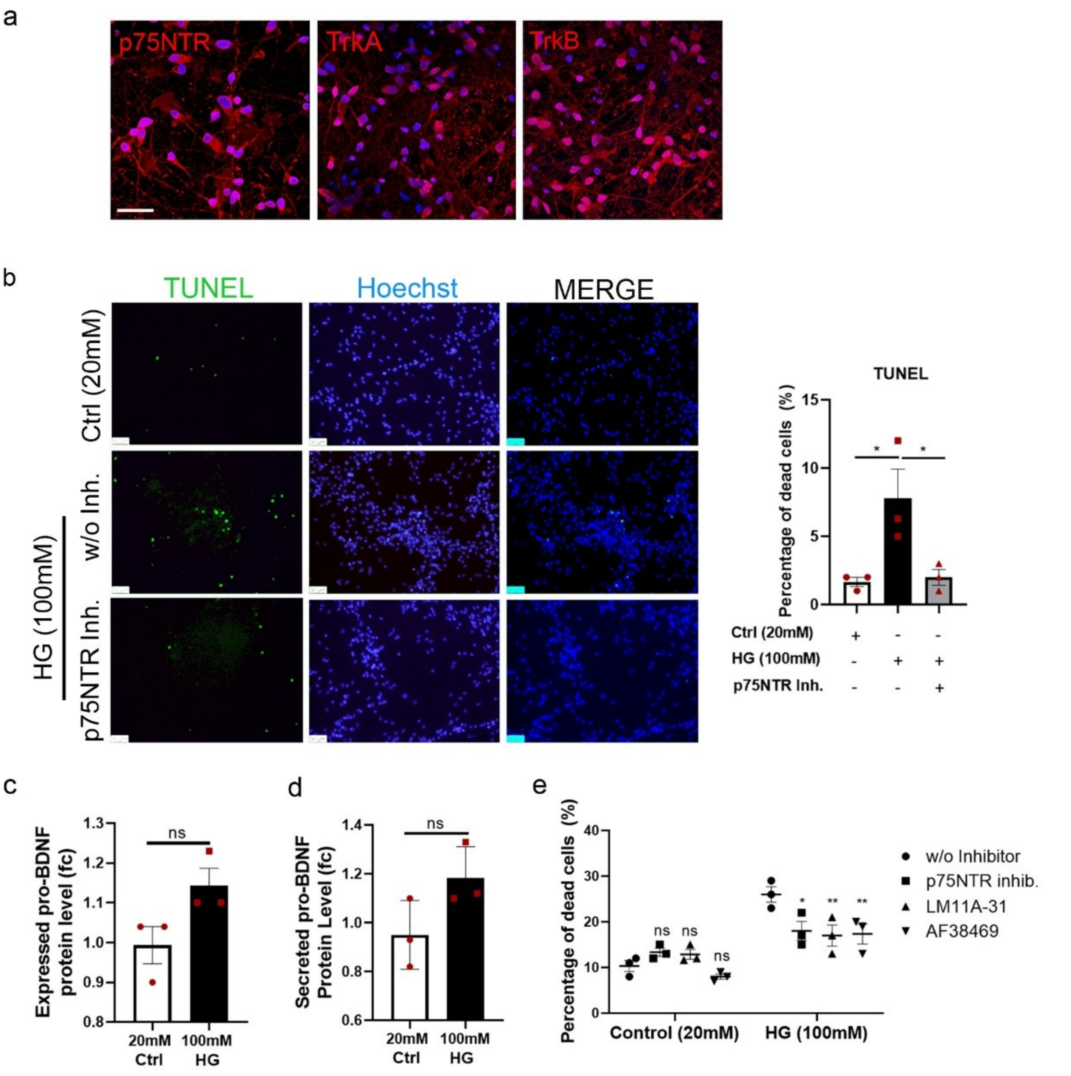

Figure S2 Analysis of p75NTR role in high glucose-treated DA neurons. a) Immunofluorescence staining of the neurotrophin receptors p75NTR, TrkA and TrkB in iPSC-derived DA neurons at differentiation day 21. Scale bar 50µm. b) TUNEL assay detecting DNA fragmentation in apoptotic cells. Representative photos and quantification of apoptotic neurons upon exposure to HG (100mM) and p75NTR Inhibitor (MC-192) for 48h. Scale bar 50µm. c,d) ELISA quantification of pro-BDNF levels in c) the cell lysate and d) the supernatant of DA neurons treated with HG (100mM) for 48hcompared to Control. e) Neuronal cytotoxicity measured by CellTox assay under control (20 mM) and high-glucose (100 mM) conditions in the presence

or absence of p75NTR inhibitor (MC-192 antibody), 400nM LM11a-31 or 1μM AF38469 Sortilin inhibitor for 48h. Data are presented as mean ± SEM of three biological replicates derived from three independent iPSC lines. For b statistical significance was assessed using ordinary one-way ANOVA with Turkey's multiple comparisons test (*P < 0.05). For c and d statistical significance was assessed using unpaired t-test (for b P=0.07, for c P=0.1). For e statistical significance was assessed using a two-way ANOVA with Dunnett's multiple comparisons test. *P < 0.05 **P < 0.01 vs respective control within each glucose condition.

Figure S3

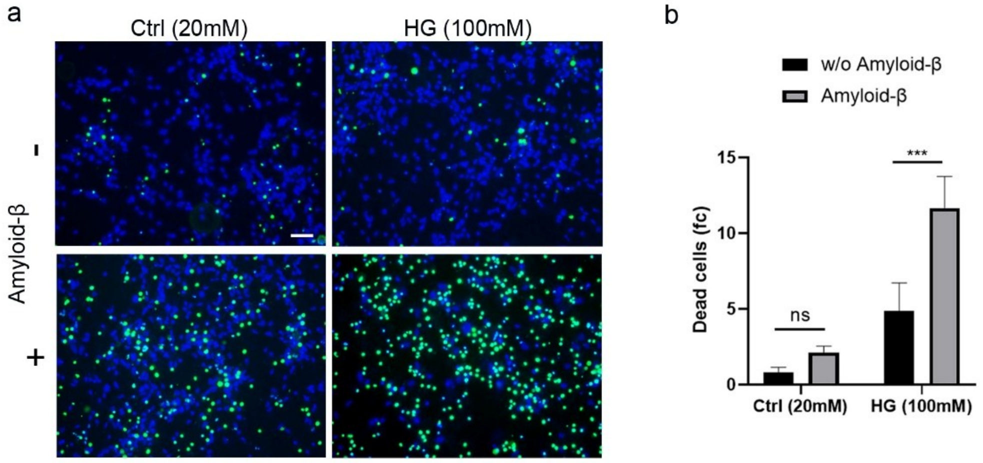

Figure S3. High glucose triggers DA neuron susceptibility to Amyloid-β 1-42. (a) Representative images and (b) quantification of dead (green) neurons upon exposure to HG (100mM) and/or 10µM oligomers of Amyloid-β 1-42 for 48 h. Scalebar 50µm. For b data are shown as mean +SEM of three biological replicates derived from three independent iPSC lines. Statistical significance was evaluated with two-way ANOVA with Sidak's multiple comparisons test (***P<0.001).

Figure S4

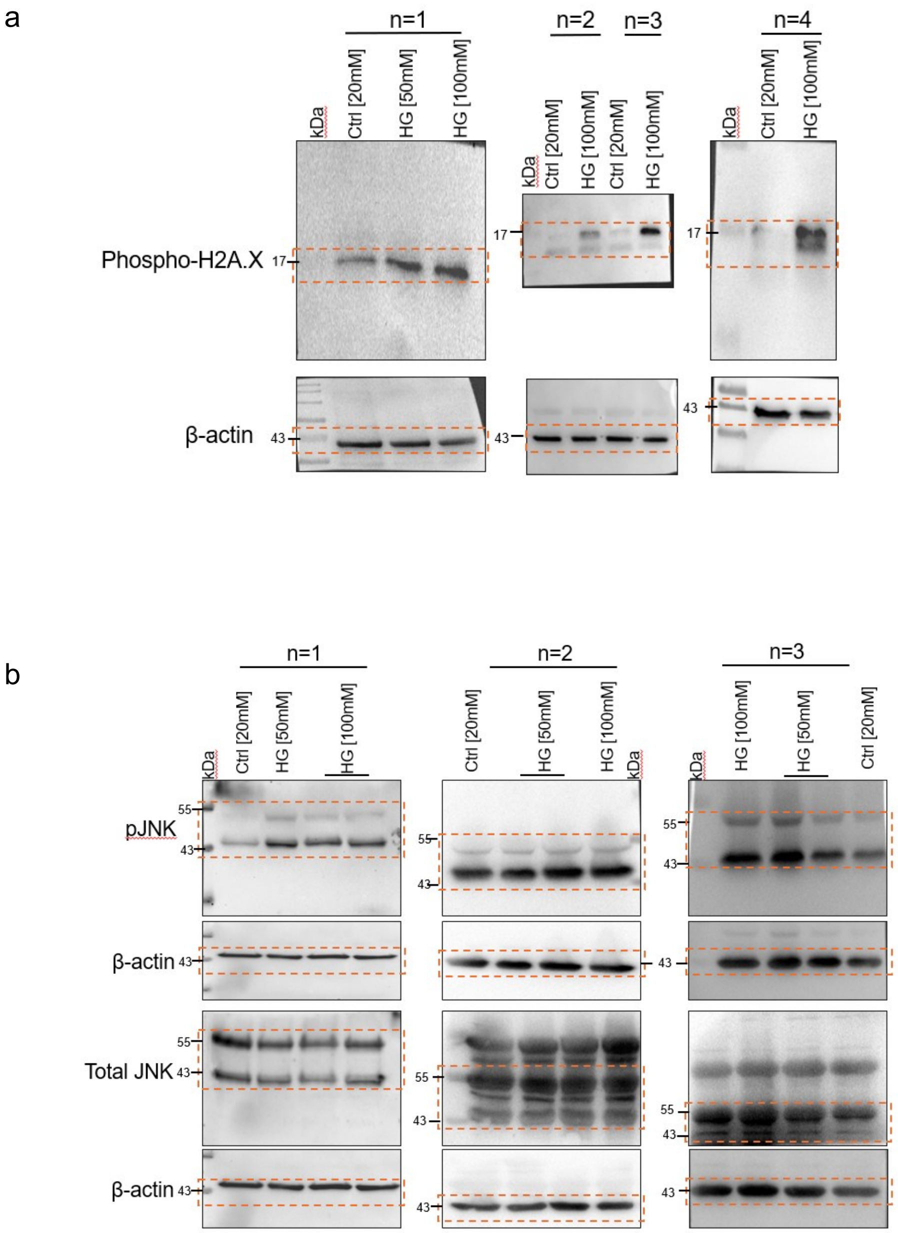

Figure S4. Corresponding full-length Western blots of Figure1d, f.

Figure S5

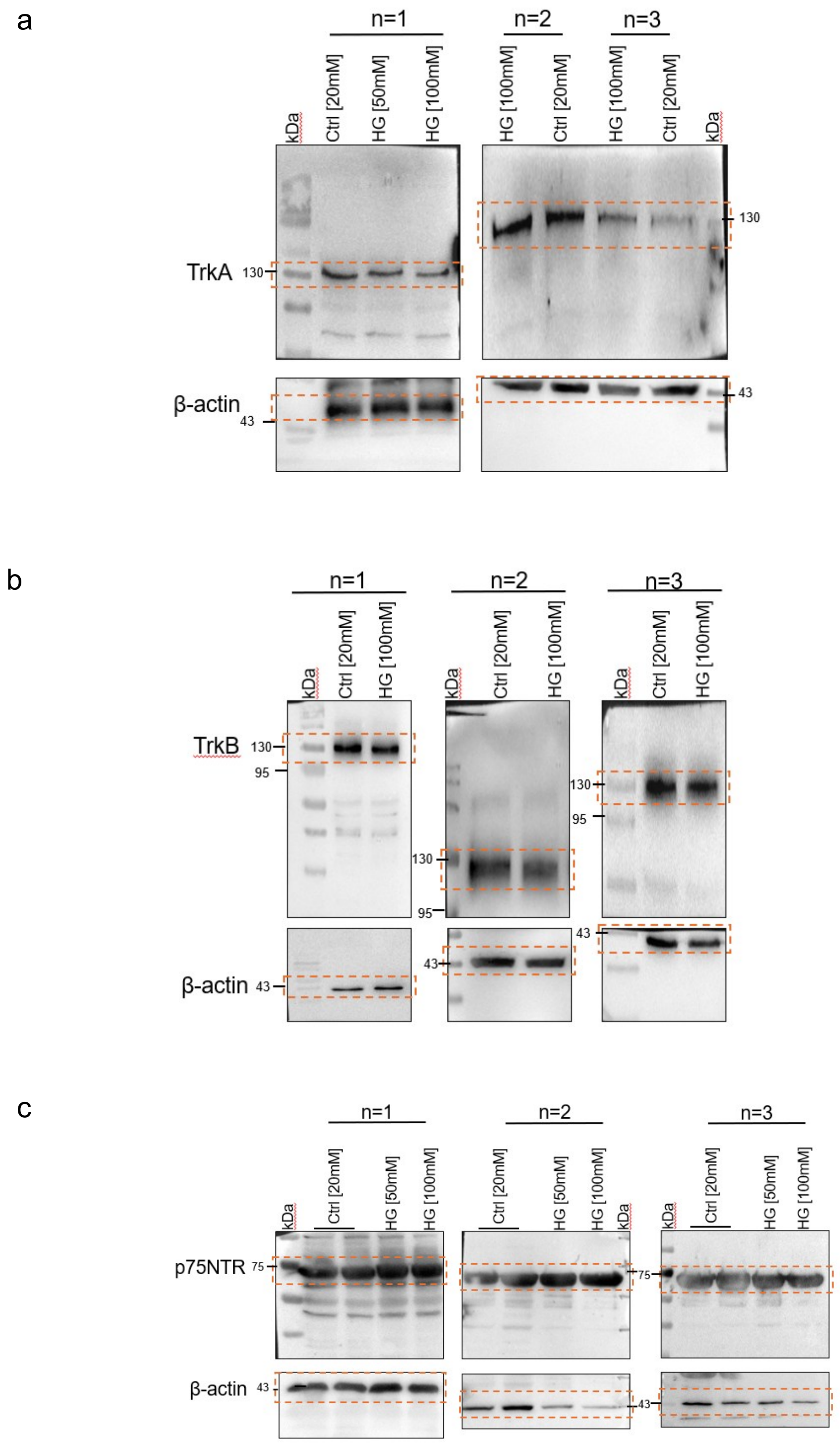

Figure S5. Corresponding full-length Western blots of Figure 3a, b.
